# Human intestinal tissue-resident memory T cells comprise transcriptionally and functionally distinct subsets

**DOI:** 10.1016/j.celrep.2020.108661

**Published:** 2021-01-19

**Authors:** Michael E.B. FitzPatrick, Nicholas M. Provine, Lucy C. Garner, Kate Powell, Ali Amini, Sophie L. Irwin, Helen Ferry, Tim Ambrose, Peter Friend, Georgios Vrakas, Srikanth Reddy, Elizabeth Soilleux, Paul Klenerman, Philip J. Allan

**Affiliations:** 1 Translational Gastroenterology Unit, Nuffield Department of Medicine, University of Oxford, Oxford OX3 9DU, UK; 2 Peter Medawar Building for Pathogen Research, University of Oxford, Oxford OX1 3SY, UK; 3 Nuffield Department of Surgical Sciences, University of Oxford, Oxford OX3 9DU, UK; 4 Oxford Transplant Centre, Churchill Hospital, Oxford University Hospitals NHS Foundation Trust, Oxford OX3 7LE, UK; 5 Department of Pathology, University of Cambridge, Tennis Court Road, Cambridge CB2 1QP, UK; 6 NIHR Biomedical Research Centre, John Radcliffe Hospital, Oxford University Hospitals NHS Foundation Trust, Oxford OX3 9DU, UK

**Keywords:** Tissue resident, residency, intestine, human, T cell, β2-integrin, CD103, transplant, intestinal transplantation

## Abstract

Tissue-resident memory T (T_RM_) cells provide key adaptive immune responses in infection, cancer, and autoimmunity. However, transcriptional heterogeneity of human intestinal T_RM_ cells remains undefined. Here, we investigate transcriptional and functional heterogeneity of human T_RM_ cells through study of donor-derived T_RM_ cells from intestinal transplant recipients. Single-cell transcriptional profiling identifies two transcriptional states of CD8^+^ T_RM_ cells, delineated by *ITGAE* and *ITGB2* expression. We define a transcriptional signature discriminating these populations, including differential expression of cytotoxicity- and residency-associated genes. Flow cytometry of recipient-derived cells infiltrating the graft, and lymphocytes from healthy gut, confirm these CD8^+^ T_RM_ phenotypes. CD8^+^ CD69^+^CD103^+^ T_RM_ cells produce interleukin-2 (IL-2) and demonstrate greater polyfunctional cytokine production, whereas β2-integrin^+^CD69^+^CD103^−^ T_RM_ cells have higher granzyme expression. Analysis of intestinal CD4^+^ T cells identifies several parallels, including a β2-integrin^+^ population. Together, these results describe the transcriptional, phenotypic, and functional heterogeneity of human intestinal CD4^+^ and CD8^+^ T_RM_ cells.

## Highlights

• Human intestinal transplants were used to identify bona fide T_RM_ cells
• Single-cell RNA sequencing identifies two distinct CD8^+^ T_RM_ subsets
• CD103^+^CD69^+^ and CD103^−^CD69^+^ T_RM_ cell subsets show distinct localization and function
• β2-integrin is highly expressed on CD103^−^ T_RM_ cells

## Graphical Abstract

**Figure:**
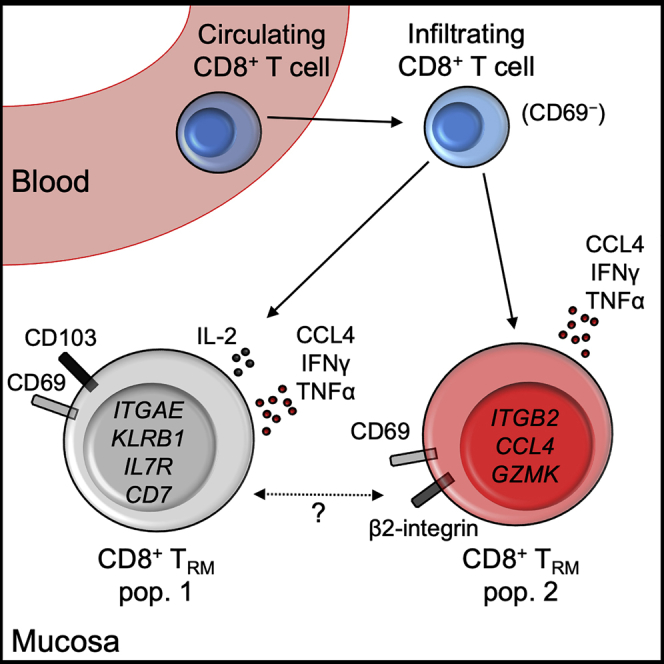

## Teaser

It has been historically difficult to accurately identify human tissue-resident memory T (T_RM_) cells. FitzPatrick et al. use a model of human intestinal transplantation to more definitively identify human T_RM_ cells. Single-cell RNA sequencing reveals two transcriptionally distinct populations of intestinal CD8^+^ T_RM_ cells, which show differences in localization and function.

## Introduction

Tissue-resident memory T (T_RM_) cells are a subset of long-lived T cells that reside in tissue and do not recirculate (Mackay and Kallies, 2017; Szabo et al., 2019). T_RM_ populations provide rapid, *in situ*, adaptive protection against a wide spectrum of pathogens (Gebhardt et al., 2011; Schenkel et al., 2014). T_RM_ cells also have key roles in cancer immune surveillance (Park et al., 2019) and are implicated in autoimmunity, including inflammatory bowel disease (IBD) and celiac disease (Mayassi et al., 2019; Zundler et al., 2019). CD8^+^ T_RM_ cells have potent cytotoxic functions and produce pro-inflammatory cytokines to trigger innate and adaptive immune responses (Ariotti et al., 2014); Schenkel et al., 2014).

Murine work has advanced our understanding of T_RM_ cells substantially; however, T_RM_ phenotype, transcriptional profiles, and genetic regulation differ between mice and humans (Hombrink et al., 2016; Kumar et al., 2017; Oja et al., 2018). Until recently, human studies of T_RM_ biology were hampered by the inability to prove long-lived tissue residency, with surface molecules CD69 and CD103 (αE-integrin) used as surrogate T_RM_ markers. These were used to identify the putative transcriptional signature of human T_RM_ cells, with CD69 hypothesized as the key surrogate marker of residency (Kumar et al., 2017). However, this gene signature was derived from bulk populations, so it remains unclear whether there are transcriptionally distinct subsets within human T_RM_ cells.

Despite its expression on almost all murine and human T_RM_ cells, the use of CD69 as a residency marker has recently been questioned. CD69 restricts lymphocyte tissue egress via S1P1 inhibition but is not required for the development of functional T_RM_ cells in mice (Shiow et al., 2006; Walsh et al., 2019). CD69 can be induced via stimulation, and a proportion of CD69^+^ T cells in tissue are not resident, making CD69 a suboptimal residency marker (Beura et al., 2018; Sancho et al., 2005; Shiow et al., 2006). Therefore, additional phenotypic markers to identify CD103^−^ T_RM_ populations are required.

Recent work has exploited the human model of organ transplantation to study long-lived, donor-derived T cells, which are definitively functionally resident T_RM_ cells (Bartolomé-Casado et al., 2019; Snyder et al., 2019; Zuber et al., 2016). Studies using this approach have demonstrated persistence of clonally identical intestinal CD8^+^ T_RM_ cells for up to 1 year in the small intestine (SI) (Bartolomé-Casado et al., 2019), and persistency of donor-derived T cells for 600 days after transplant (Zuber et al., 2016). This approach was also used to identify putative SI CD8^+^ T_RM_ cell subsets based on expression of CD103 and KLRG1, with differences in clonality, granzyme expression, and cytokine production (Bartolomé-Casado et al., 2019). However, it remains unclear whether these cell populations represent transcriptionally distinct subsets.

This work sought to examine the heterogeneity within functionally resident, donor-derived T cells in intestinal transplantation using flow cytometry and single-cell RNA sequencing (scRNA-seq). We confirmed that human SI CD4^+^ and CD8^+^ T_RM_ cells can persist for 5 years after transplant. scRNA-seq identified conventional and regulatory CD4^+^ T_RM_ cell populations, as well as two transcriptionally distinct CD8^+^ T_RM_ subsets, which differed in expression of *ITGAE* (CD103, αE-integrin) and *ITGB2* (CD18, β2-integrin). These two populations differentially expressed putative T_RM_-associated genes, indicating that the gene signatures derived from bulk RNA sequencing (RNA-seq) data may be a synthesis of several transcriptomic profiles. We validated this phenotypic and functional heterogeneity in the healthy intestine, with CD103^−^ T_RM_ cells showing increased β2-integrin expression and distinctive effector function. CD69, β2-integrin, and CD103 expression changed with time post-transplant on recipient-derived, graft-infiltrating CD8^+^ T cell populations, consistent with acquisition of T_RM_ status. We conclude that CD69^+^CD103^−^β2-integrin^+^ CD8^+^ intestinal T cells are a transcriptionally and functionally distinct T_RM_ cell population and suggest that β2-integrin can serve as an adjunct surface marker to CD69 for CD103^−^ T_RM_ cells.

## Results

### Long-lived, conventional CD4^+^ and CD8^+^ T cells, but not unconventional T cell populations, can persist for at least 5 years in the human intestine

**Figure 1:**
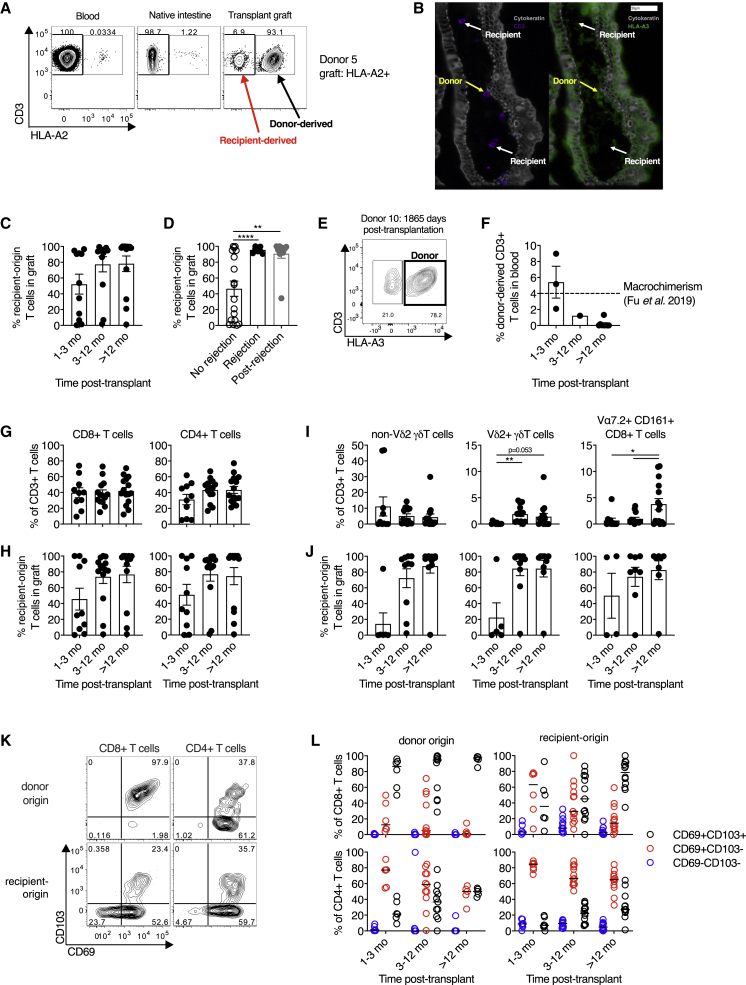
Long-lived, conventional CD4^+^ and CD8^+^ T cell, but not unconventional T cell, populations can persist for at least 5 years in the human intestine (A) Representative flow cytometry plot of HLA-A2 expression on T cells from the blood, the recipient native intestinal mucosa, and the intestinal transplant graft demonstrating identification of donor- and recipient-derived populations by HLA mismatch. (B) Representative chip cytometry image of intestinal graft mucosa demonstrating the presence of donor-derived (HLA-A3^+^, yellow arrows) and recipient-derived (HLA-A3^−^, white arrows) CD3^+^ T cells in the lamina propria. Cytokeratin (gray); CD3 (purple); HLA-A3 (green). (C) Percentage of recipient-origin CD3^+^ T cells in intestinal grafts, categorized by time after transplant (n = 37; 16 subjects; means ± SEM). (D) Percentage of recipient-origin CD3^+^ T cells in intestinal grafts, categorized by history of graft rejection (n = 37; 16 subjects; means ± SEM). (E) Flow cytometry plot of HLA-A3 expression on graft-derived T cells in one subject who demonstrated persistent donor chimerism in the intestinal graft 1,865 days (5 years and 1 month) after transplant. (F) Percentage of donor-origin CD3^+^ T cells in the blood of intestinal transplant recipients, categorized by time after transplant (n = 13; means ± SEM). Dashed line at 4% represents the cutoff for significant macrochimerism from prior studies (Fu et al., 2019; Zuber et al., 2015). (G) Conventional CD8^+^ and CD4^+^ T cell subsets in the small intestinal graft as a proportion of total T cells, categorised by time post-transplant (n = 39; 18 subjects; means ± SEM). (H) The percentage of recipient-derived T cells within conventional CD8^+^ and CD4^+^ T cell subsets in the intestinal graft, categorized by time after transplant (n = 37; 16 subjects; means ± SEM). (I) Unconventional non-Vδ2^+^ γδ T cell, Vδ2^+^ γδ T cell, and Vα7.2^+^CD161^+^ CD8^+^ T cell (mucosal-associated invariant T cell) subsets in the small intestinal graft as a proportion of total T cells, categorized by time after transplant (n = 39; 18 subjects; means ± SEM). (J) The percentage of recipient-derived T cells infiltrating the intestinal graft within unconventional non-Vδ2^+^ γδ T cell (n = 27; 12 subjects), Vδ2^+^ γδ T cell (n = 25; 12 subjects), and Vα7.2^+^CD161^+^ CD8^+^ T cell (n = 20; 12 subjects) subsets, categorized by time after transplant (means ± SEM). (K) Representative flow cytometry plot of CD69 and CD103 expression on donor- and recipient-derived CD8^+^ and CD4^+^ T cells in the intestinal graft. (L) Percentage of donor- and recipient-derived CD8^+^ and CD4^+^ T cells in the intestinal graft co-expressing CD69 and CD103 categorized by time after transplant (n = 35; 16 subjects; median marked with black line). For further analysis of surface marker expression, rare populations with fewer than 10 cells were excluded from the analysis (J and L). Statistical analysis performed with one-way ANOVA with Dunnett’s multiple-comparison test. ^∗^p ≤ 0.05, ^∗∗^p ≤ 0.01.

To examine the persistence of resident T cells in the SI after transplant, we used human leukocyte antigen (HLA) allele congenic cell tracking, a method allowing discrimination of donor- and recipient-derived cells after transplantation using fluorophore-conjugated antibodies to discordant class I HLA haplotypes (Figures 1A and S1A) (Bartolomé-Casado et al., 2019; Zuber et al., 2016). The presence of SI donor- and recipient-derived T cells *in situ* was confirmed by chip cytometry (Figure 1B) (Leng et al., 2019).

Infiltration of recipient-derived T cells into the graft increased over time, with striking heterogeneity (Figure 1C). Current or previous rejection was associated with higher proportions of recipient-derived T cells in the graft (Figure 1D), consistent with prior work (Zuber et al., 2016). Conversely, two individuals had persistent, donor-derived SI T cell populations at 4 years 7 months (1,684 days) and 5 years 1 month (1,865 days) after transplant (Figure 1E and data not shown). This extends previous reports of long-lived donor chimerism in the human intestine (Bartolomé-Casado et al., 2019; Zuber et al., 2016).

Hematopoietic stem and progenitor cells can persist in the intestinal transplant graft, sometimes leading to long-term chimerism of donor-derived populations in blood (Fu et al., 2019). This could potentially allow persistence of donor-derived T cells in the graft through continuous ingress from blood, rather than via residency (Bartolomé-Casado et al., 2019). Contrasting with the work of Fu et al. (2019), flow cytometric analysis of blood collected from transplant recipients at the time of biopsy revealed the percentage of circulating donor origin T cells was extremely low beyond 3 months after transplant (median, 0.042%; 95% confidence interval [95% CI], 0%–1.22%; Figure 1F). This is far below the posited cutoff for macrochimerism of 4% (Fu et al., 2019) and is similar to background, non-specific staining in non-transplant recipients (0.01%–0.12%; Figure S1B), indicating that continuous replacement is unlikely to be a confounding mechanism for sustained donor chimerism in our cohort. This discrepancy in circulating chimerism between studies may be due to differences in transplant procedure (multi-visceral versus isolated intestinal transplant in this study) and recipient age (pediatric versus adult in this study) (Fu et al., 2019; Zuber et al., 2015).

Conventional CD4^+^ and CD8^+^ T cells dominated in the graft after transplant (Figure 1G) and demonstrated similar kinetics of increased recipient-derived cells over time (Figure 1H). We also examined the dynamics of unconventional T cell subsets after transplant because their residency characteristics in humans are poorly understood (Figure S1C). CD161^+^Vα7.2^+^ CD8^+^ T cells (consistent with mucosal-associated invariant T [MAIT] cells) and Vδ2^+^ γδ T cells (which possess analogous innate-like functions to MAIT cells [Gutierrez-Arcelus et al., 2019; Provine et al., 2018]) were rare in the graft after transplant, recovering to expected frequencies after 1 year (Figure 1I). However, the non-Vδ2 γδ T cell subsets demonstrated different dynamics, with this population present at early time points. Unconventional T cell subsets demonstrated similar replacement kinetics (Figure 1J). It is unclear whether the low innate-like T cell frequency post-transplant is due to differences in residency characteristics or to increased sensitivity to the ischemic insult of surgery or perioperative conditioning regimes.

We examined CD69 and CD103 expression on donor- and recipient-derived CD4^+^ and CD8^+^ T cell populations in the intestinal graft. CD103 expression was restricted to CD69^+^ cells, with a greater proportion of CD8^+^ cells expressing CD103 than CD4^+^ cells (Figure 1K), in keeping with prior work (Bartolomé-Casado et al., 2020; Kumar et al., 2017). Donor-derived T cells showed near-ubiquitous expression of CD69 consistent with a lack of recent migration from blood and comprised fewer CD69^−^ cells than recipient-derived populations for both CD8^+^ (median 0.03% versus 4.17%, p < 0.0001, Wilcoxon signed-rank test) and CD4^+^ (median 0.10% versus 5.65%, p < 0.0001, Wilcoxon signed-rank test) cells (Figure 1L). Although recent murine data showed no functional requirement for CD69 to establish intestinal residency, these data suggest that lack of CD69 expression on intestinal T cells is a good surrogate for definitively non-resident populations (Walsh et al., 2019). Recipient-derived CD4^+^ and CD8^+^ T cell populations showed increasing expression of CD103 with time, consistent with the acquisition of a T_RM_ phenotype, as in prior work (Zuber et al., 2016). We have previously shown higher expression of the C-type, lectin-like receptor CD161 on intestinal CD103^+^ CD8^+^ T cells (Fergusson et al., 2016); here, a greater proportion of donor-derived CD8^+^ T cells expressed CD161, consistent with an association with residency (Figures S1D and S1E).

### scRNA-seq delineates transcriptionally distinct states within CD4^+^ and CD8^+^ T_RM_ cell populations

**Figure 2:**
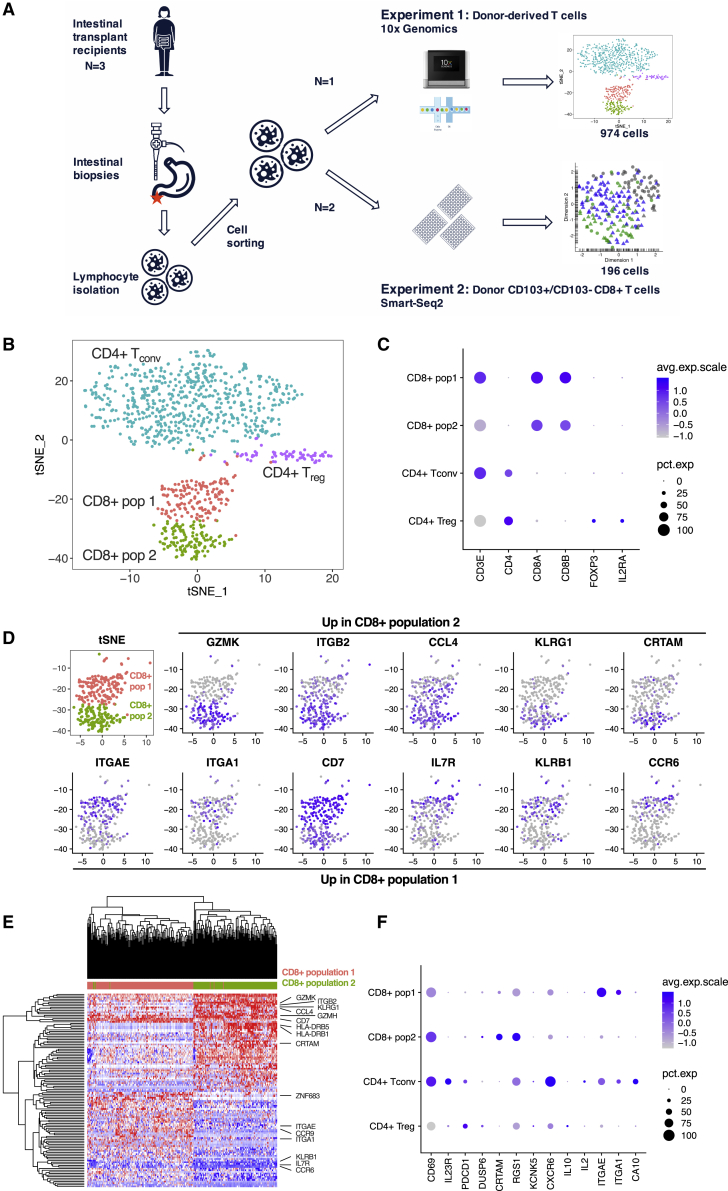
Single-cell RNA sequencing (scRNA-seq) delineates transcriptionally distinct states within CD4^+^ and CD8^+^ T_RM_ cell populations (A) Schematic of two scRNA-seq experiments. Biopsies of small intestinal transplant tissue were collected from subjects at endoscopy, then dissociated to isolate intestinal lymphocytes. These were sorted by fluorescence-activated cell sorting (FACS), first with bulk sorting of donor-derived T cells in experiment 1, then with index sorting of donor-derived CD103^−^ and CD103^+^ T cells in experiment 2, before scRNA-seq library preparation and sequencing using the 10× Genomics platform (experiment 1) or the Smart-Seq2 protocol (experiment 2). (B) t-Distributed Stochastic Neighbor Embedding (tSNE) plot of 974 donor-derived T cells from a single subject showing four transcriptionally distinct populations of conventional CD4^+^ and CD8^+^ T cells. (C) Dot plot of key gene identifiers for the four clusters showing two clusters of CD8^+^ T cells and two clusters of CD4^+^ T cells, including one containing cells expressing *IL2RA* and *FOXP3*, consistent with a regulatory T cell phenotype. Dot size indicates the proportion of cells in which the gene is expressed. (D) tSNE plots showing the expression of key genes upregulated in CD8^+^ population 2 (top row) or upregulated in CD8^+^ population 1 (bottom row). (E) Heatmap indicating hierarchical clustering of gene expression of CD8^+^ donor-derived T cells from population 1 and population 2. Cell labels above indicate cluster: red, population 1; green, population 2. Genes of interest are highlighted. (F) Dot plot showing the expression of 13 genes previously associated with tissue residency in human CD69^+^ T cells (Kumar et al., 2017). Dot size indicates the proportion of cells in which the gene is expressed.

The persistence of CD103^−^ and CD103^+^ donor-derived T cells up to 5 years after transplant, and the enrichment of CD103^+^ recipient-derived T cells at later time points, raised the possibility that CD103^−^ and CD103^+^ T cells represented distinct cell states. To test that hypothesis, we performed droplet-based scRNA-seq of sorted donor-derived, graft-resident T_RM_ cells from a single subject 1 year after transplant (experiment 1; Figure 2A); 1,774 cells were captured and sequenced, with 974 cells remaining after filtering (Figure S2A).

Four transcriptionally distinct clusters were identified (Figure 2B): conventional CD4^+^ T cells, regulatory CD4^+^ T cells expressing *IL2RA* and *FOXP3*, and two clusters of conventional CD8^+^ T cells (Figure 2C). The differentially expressed genes (DEGs) between the two populations of donor-derived CD8^+^ T_RM_ cells (hereafter, CD8^+^ populations 1 and 2) were analyzed (Figure 2D). Population 1 expressed *ITGAE* (CD103), as well as more *CD7*, *IL7R* (CD127), *KLRB1* (CD161), and the chemokine receptor *CCR6* (Figures 2D and 2E). IL-7, a stromal-derived homeostatic cytokine that provides survival and proliferative signals to lymphocytes (Raeber et al., 2018), is required for epidermal T_RM_ cell persistence (Adachi et al., 2015), and *CD127* is highly expressed in SI memory T cells (Thome et al., 2014). The differential expression of *IL7R* suggests differences in the mechanisms, or nature, of T_RM_ cell persistence between the two populations (Raeber et al., 2018).

Conversely, CD8^+^ population 2 expressed low levels of *ITGAE* but high levels of *KLRG1*, as well as cytotoxic granzyme molecules and class II major histocompatibility complex (MHC) molecules (Figures 2D and 2E). Surface KLRG1 expression can delineate two putative SI CD103^−^ CD8^+^ T cell subsets with similar residency characteristics (Bartolomé-Casado et al., 2019). However, population 2 did not sub-cluster further, based on *KLRG1* gene expression, suggesting that these are not transcriptionally distinct states. Of particular interest, the integrin *ITGB2* was highly expressed by the CD103^−^ CD8^+^ population 2. β2-integrin can form heterodimers with four α-integrins (Fagerholm et al., 2019); only one of which, *ITGAL* (CD11a), was detected in the dataset. *ITGAL* was highly expressed on the CD103^−^ CD8^+^ population 2 (Figures S2B and S2C).

### CD8^+^ CD103^+^CD69^+^ and CD103^−^CD69^+^ ITGB2^hi^ T_RM_ cell subsets differ in expression of putative residency-associated genes

Transcriptional signatures associated with human tissue residency have been defined, most thoroughly by bulk RNA-seq of CD69^−^ and CD69^+^ T cells from multiple tissues (Kumar et al., 2017). This T_RM_ cell gene set was explored in the two CD8^+^ T cell populations. As expected, the genes downregulated in CD69^+^ T cells were either not detected or were found at low levels in both clusters (Figures S2D and S2E). However, several T_RM_ cell-associated genes upregulated in CD69^+^ T cells were differentially expressed between the two clusters, with population 1 expressing more *ITGAE* and *ITGA1* (in agreement with work on renal T_RM_ cells [de Leur et al., 2019]), and population 2 expressing more *CRTAM* (Figures 2D, 2F, and S2F). T cell receptor (TCR) repertoire analysis revealed the presence of shared clonotypes between the two T_RM_ clusters (Figure S2G). These data indicate that previously identified T_RM_ cell gene signatures may represent an amalgamation of several distinct T_RM_ cell transcriptional states.

### scRNA-seq identifies a core gene set distinguishing two CD8^+^ T_RM_ cell populations

**Figure 3:**
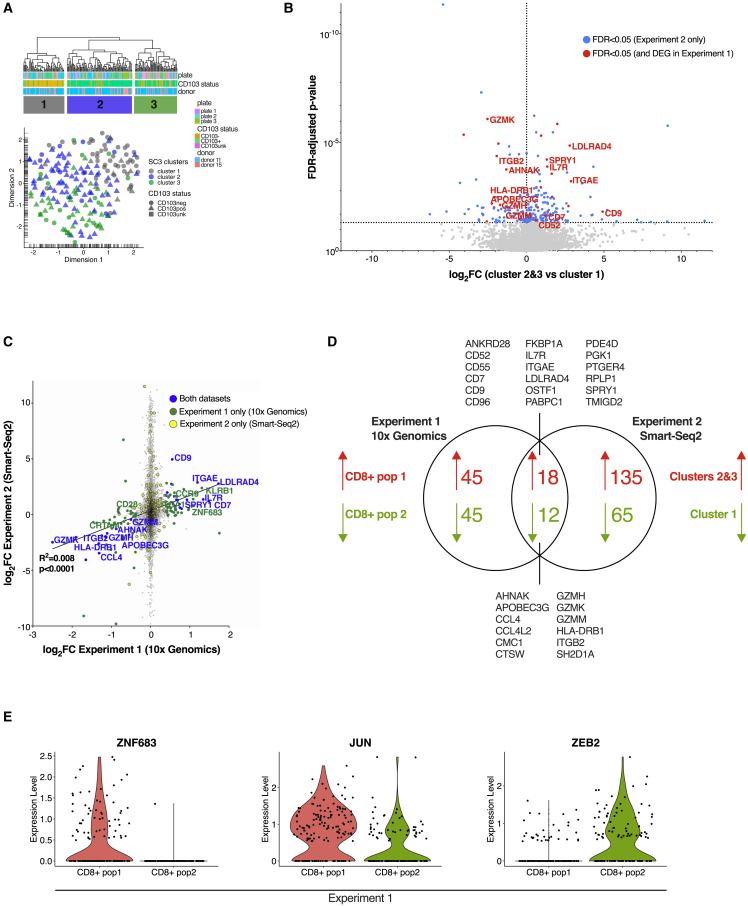
Single-cell RNA sequencing identifies a core gene set distinguishing two CD8^+^ T_RM_ cell populations (A) Hierarchical clustering and Uniform Manifold Approximation and Projection in R (UMAP) plot of 196 index-sorted CD103^−^ and CD103^+^ CD8^+^ donor-derived T cells from two subjects in experiment 2 identified three transcriptionally distinct clusters of conventional CD8^+^ T cells. Cell labels below the dendrogram indicate the sorting plate, the subject, and CD103 expression determined by index sorting. For UMAP plot: circles, CD103^−^ cells by index sorting; triangles, CD103^+^ cells by index sorting; square, unknown CD103 expression (n = 1 cell) by index sorting. (B) Volcano plot of differential gene expression between cluster 1 and clusters 2 + 3 identified in experiment 2, with genes upregulated (right) or downregulated (left) in clusters 2 + 3 compared with cluster 1. Log_2_ fold change is plotted against the false discover rate (FDR)-adjusted p-value, with horizontal dotted line at FDR = 0.05. Differentially expressed genes are marked in blue, with those differentially expressed in both experiments 1 and 2 marked in red. (C) Correlation of log_2_ fold change in CD8^+^ population 1 versus 2 in experiment 1 (10× Genomics, x axis) and clusters 2 + 3 versus cluster 1 in experiment 2 (Smart-Seq2, y axis). Blue, genes differentially expressed in both experiments; green, genes differentially expressed in experiment 1 only; yellow, genes differentially expressed in experiment 2 only; gray, genes not differentially expressed. (D) Venn diagram showing differentially expressed genes upregulated in clusters 2 + 3 (experiment 2)/CD8^+^ population 1 (experiment 1) (red) versus genes upregulated in cluster 1 (experiment 2)/CD8^+^ population 2 (experiment 1) (green). A core gene set of 30 genes that differentiated the two populations in both experiments is listed. (E) Violin plots showing expression of transcription factors *ZNF683*, *JUN*, and *ZEB2*, which showed differential expression between CD8^+^ T_RM_ cell populations in experiment 1.

To confirm the presence of transcriptionally distinct CD8^+^ T_RM_ cell states, a second scRNA-seq experiment was performed on samples from two further transplant recipients (experiment 2; Figure 2A). Donor-derived CD103^−^ and CD103^+^ CD8^+^ T cells were index sorted before plate-based scRNA-seq using the Smart-Seq2 protocol (Picelli et al., 2013); 267 cells were sorted and sequenced, with 196 cells remaining after filtering (Figures S2H–S2J). Three clusters were identified, with cluster 1 predominantly formed of CD103^−^ T cells and the transcriptionally similar clusters 2 and 3 formed of CD103^+^ T cells (Figure 3A). Clusters 2 and 3 (CD103^+^) expressed more *ITGAE*, *CD7*, and *IL7R*, whereas cluster 1 (CD103^−^) expressed more *GZMK*, *GZMH*, class II HLA molecules, and *ITGB2* (Figure 3B). *ITGAL* was also detected in cluster 1 (Figure S2K).

Differential expression analysis between cluster 1 and clusters 2 + 3 revealed congruent transcriptional differences to those in experiment 1 (Figure 3C; Tables S2 and S3). Comparison of DEGs in the two experiments identified a transcriptional signature of 30 genes that distinguished the two CD8^+^ T_RM_ populations (Figures 3C and 3D).

### Transcription factor expression in T_RM_ subsets

We examined expression of transcription factors (TFs), identified from Lambert et al. (2018), in the two CD8^+^ clusters in experiment 1. *ZNF683* (Hobit), a transcriptional regulator of residency in mice (Mackay et al., 2016), but which was not linked with a human T_RM_-associated gene set (Kumar et al., 2017), was more highly expressed in population 1 (Figure 3E). Population 1 also showed greater expression of *JUN*, whereas population 2 expressed more *ZEB2*, a TF associated with terminally differentiated effector CD8^+^ T cell populations (Omilusik et al., 2015). Other TFs previously associated with tissue residency were not differentially expressed between the CD8^+^ T_RM_ cell populations, although *PRDM1* and *BHLHE40* showed increased expression in the CD4^+^ T_conv_ T_RM_ cluster (Figure S3A). TF regulons (gene sets predicted to be regulated by a particular TF [Aibar et al., 2017; Van de Sande et al., 2020]) showed distinct patterns between clusters (Figure S3B; Table S4). As expected, the FOXP3 regulon was strongly associated with the T_reg_ cluster (Figures S3B and S3C). Both RUNX3 and NR4A1 regulons showed enhanced activity in both CD8^+^ T_RM_ cell clusters; these two TFs have previously been linked with residency in murine studies (Boddupalli et al., 2016; Milner et al., 2017). The module of E2F3, a TF associated with proliferative capacity, showed enhanced activity in CD8^+^ population 2, whereas PRDM1 and BHLHE40 regulons were associated with CD4^+^ T_conv_ T_RM_ cells, consistent with the increased expression of these TFs in those cells.

### CD8^+^ T_RM_ cell gene sets show differential expression in human and murine CD8^+^ intestinal T cell populations from published datasets

We sought to confirm whether these two transcriptionally distinct T_RM_ populations were seen outside the transplant setting. First, we examined the expression of our transcriptional signature distinguishing CD8^+^ T_RM_ cell populations in published scRNA-seq data from the human colon (Corridoni et al., 2020). Unsupervised hierarchical clustering of conventional memory T cell populations identified by the authors (using all DEGs) broadly divided the clusters into two groups (Figure S4A). The same clustering approach, using only our 30-gene T_RM_ cell subset transcriptional signature (Figure 3D), largely reproduced the same clustering and identified distinct populations that broadly aligned with our CD8^+^ population 1 (T_RM_, intraepithelial lymphocytes [IELs], IL26^+^, and memory) and population 2 (FGFBP2^+^, GZMK^+^ (1), and GZMK^+^ (2)) (Figure S4B). We then examined the expression of the T_RM_ cell subset transcriptional signature in three CD8A^+^/CD8B^+^ T cell clusters in published scRNA-seq data from the human ileum (Martin et al., 2019). Hierarchical clustering split genes into those associated with the two populations, with two ileal CD8^+^ clusters (4 and 47) showing similarities to CD8^+^ population 1 and cluster 21 showing similarities to CD8^+^ population 2 (Figure S4C).

Recent murine work has described transcriptionally distinct subsets of intestinal CD8^+^ T_RM_ cells in the lymphocytic choriomeningitis virus (LCMV) model of tissue residency, which highlighted CD28 as a possible marker for these subsets (Kurd et al., 2020). CD28 was increased in CD8^+^ population 2 in experiment 1, although that difference was not replicated in experiment 2 (data not shown). We examined the expression of murine orthologs of the population 2 signature in the published data. Population 2-associated genes were significantly enriched in one of the clusters at both the day 60 and 90 time points after LCMV infection, providing evidence of analogous T_RM_ cell subsets in the human and mouse intestine (Figure S4D). In sum, the CD8^+^ T_RM_ cell transcriptional signatures identified in the current study can be validated in distinct intestinal CD8^+^ T cell populations from mice and humans.

### CD103^+^ and CD103^−^ CD8^+^ T cells display distinct phenotypes in the healthy intestine

**Figure 4:**
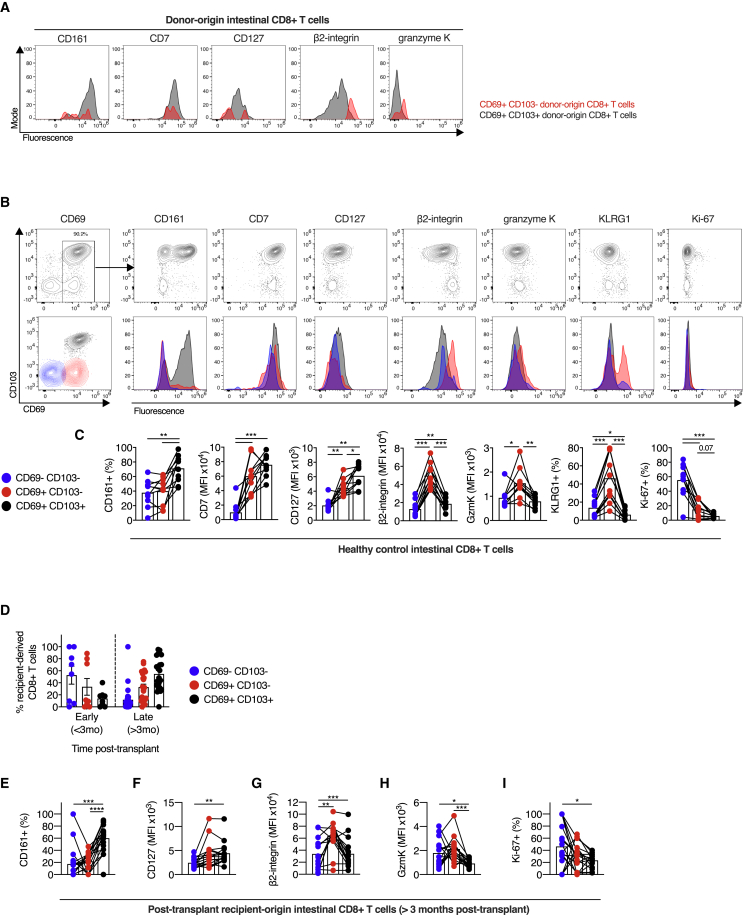
CD103^+^ and CD103^−^ CD8^+^ T cells display distinct phenotypes in healthy and transplanted intestine (A) Representative expression of CD161, CD7, CD127 (IL7R), β2-integrin (ITGB2), and granzyme K on CD103^−^ (red) and CD103^+^ (black) donor-derived T cells from the intestinal transplant graft. (B and C) Phenotypic analysis of CD8^+^ T cell populations in healthy small intestine. (B) Representative flow cytometry plots and histograms of CD8^+^ T cell phenotype from spectral flow cytometry, with expression of the following markers plotted against CD103: CD69, CD161, CD7, CD127, β2-integrin, granzyme K, KLRG1, and Ki-67. Blue, CD69^−^CD103^−^ cells; red, CD69^+^CD103^−^ cells; black, CD69^+^CD103^+^ cells. (C) Proportion of positive cells (CD161, KLRG1, and Ki-67) or MFI (CD7, CD127, β2-integrin, and granzyme K) of CD8^+^ T cells, categorized by CD69 and CD103 expression, in small intestinal biopsies from healthy control subjects (n = 10). Mean percentage or MFI represented by bars. Connecting lines represent populations from the same subject. (D–I) Phenotypic analysis of recipient-derived CD8^+^ T cell populations infiltrating the intestinal transplant graft. (D) The proportion of recipient-derived CD8^+^ T cells co-expressing CD69 and CD103 in intestinal transplant grafts, categorized by time after transplant (n = 35; 16 subjects). (E–I) Proportion of CD161^+^ cells (E), MFI of CD127 (F), β2-integrin (G), and granzyme K (H), or proportion of Ki-67^+^ cells (I) of recipient-derived CD8^+^ T cells, categorized by CD69 and CD103 expression and time after transplant in intestinal transplant grafts (n = 23; 12 subjects). Mean percentage or MFI represented by bars. Black lines connect populations from the same subject. Statistical analysis performed with one-way ANOVA with Tukey’s multiple-comparison test. ^∗^p ≤ 0.05, ^∗∗^p ≤ 0.01, ^∗∗∗^p ≤ 0.001, ^∗∗∗∗^p ≤ 0.0001.

Flow cytometry of donor-derived T cells demonstrated differences in the expression of CD161, β2-integrin, and granzyme K between CD103^−^ and CD103^+^ CD8^+^ T cells, consistent with our scRNA-seq data (Figure 4A). To validate the phenotypic differences between the two putative SI CD8^+^ T_RM_ cell subsets outside the transplant setting, we performed flow cytometry of SI T cells from healthy donors. CD8^+^ SI T cells expressing both CD69 and CD103 predominated, representing 88.9% (range, 81.7%–96.0%) of CD8^+^ T cells, with no difference observed dependent on SI location (Figure S5A).

We examined the protein expression of key markers that were differentially expressed between the two T_RM_ cell clusters in our scRNA-seq datasets. CD103^+^ CD8^+^ T cells expressed more CD161 and CD127 (IL-7R) compared with the CD69^−^ or CD69^+^CD103^−^ cells, consistent with the transcriptomic data (Figures 4B and 4C). CD7 expression was greater on all CD69^+^ T cells, with no difference between CD69^+^CD103^−^ and CD69^+^CD103^+^ populations. In contrast, CD69^+^CD103^−^ CD8^+^ T cells expressed higher levels of β2-integrin, granzyme K, and KLRG1 than either CD69^−^ cells or CD69^+^CD103^+^ cells.

Ki-67 expression formed a gradient between the three populations, with greater expression in the CD69^−^ population, and a trend toward increased Ki-67 expression in CD69^+^CD103^−^ CD8^+^ T cells compared with CD69^+^CD103^+^ CD8^+^ T cells (mean, 14.88% versus 5.72%, one-way ANOVA with Tukey’s multiple-comparison test, p = 0.067). This is consistent with prior work indicating that T_RM_ cell persistence is due to longevity, rather than to *in situ* proliferation (Thome et al., 2014).

### Graft-infiltrating, recipient-derived T cells take on a T_RM_ cell phenotype over time, with CD103^+^ and CD103^−^ CD8^+^ T cells displaying distinct phenotypes

To explore the dynamics of T_RM_ cell phenotype acquisition, we examined graft-infiltrating, recipient-derived T cells. In the early post-transplant period, 52.7% of infiltrating CD8^+^ T cells lacked CD69 expression, with acquisition of the CD69^+^CD103^+^ T_RM_ cell phenotype over time (Figure 4D). At later times post-transplant, CD103^−^ and CD103^+^ populations clearly differed in phenotype, consistent with the two subsets observed in the healthy SI. CD161 expression was greater on CD69^+^CD103^+^ CD8^+^ T cells than CD69^+^CD103^−^ CD8^+^ T cells, and granzyme K expression was greater on CD69^+^CD103^−^ CD8^+^ T cells (Figures 4E–4I). Graft-infiltrating CD69^+^CD103^−^ CD8^+^ T cells demonstrated higher expression of β2-integrin than either CD69^−^ or CD69^+^CD103^+^ populations (Figure 4G), while CD69^+^ populations showed higher Ki-67 expression as before (Figure 4I). Recipient-derived CD8^+^ T cells infiltrating the graft in the early post-transplant period displayed some analogous patterns of expression, including of β2-integrin, although the small number of samples precluded further analysis (Figure S5B).

### CD103^+^ and CD103^−^ CD8^+^ T cells maintain their distinct phenotypes in lamina propria and intra-epithelial compartments

**Figure 5:**
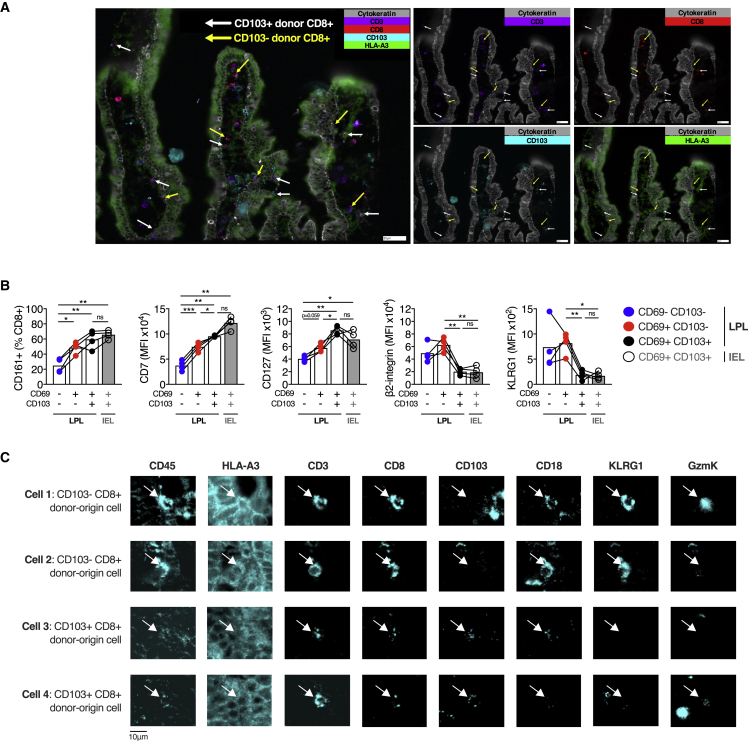
Graft-infiltrating, recipient-derived T cells take on a T_RM_ cell phenotype over time, with CD103^+^ and CD103^−^ CD8^+^ T cells displaying distinct phenotypes (A) Fluorescence microscopy chip cytometry image of small intestinal transplant mucosa from a single subject 3 months after transplant. The donor was HLA-A3^+^, and the recipient was HLA-A3^−^. False-color fluorescence imaging for cytokeratin (gray), CD3 (purple), CD8 (red), CD103 (blue), and HLA-A3 (green). Donor-derived CD8^+^ CD103^+^ and CD103^−^ T cells are indicated by white and yellow arrows, respectively. (B) Phenotypic analysis of CD8^+^ T cell populations in healthy small intestinal epithelium and lamina propria. Proportion of positive cells (CD161) or MFI (CD7, CD127, β2-integrin, and KLRG1) of intraepithelial lymphocyte (IEL) or lamina propria lymphocyte (LPL) CD8^+^ T cells, categorized by CD69 and CD103 expression, in small intestinal biopsies from healthy control subjects (n = 4). Mean percentage or MFI represented by bars. Connecting lines represent populations from the same subject. (C) Fluorescence microscopy chip cytometry of representative CD103^−^ (cells 1 and 2) and CD103^+^ (cells 3 and 4) donor-derived CD8^+^ T cells, showing the expression of CD18 (β2-integrin), KLRG1, and granzyme K. Statistical analysis performed with one-way ANOVA with Tukey’s multiple-comparison test. ^∗^p ≤ 0.05, ^∗∗^p ≤ 0.01, ^∗∗∗^p ≤ 0.001, ^∗∗∗∗^p ≤ 0.0001.

We examined the localization of these identified populations within the intestinal mucosa. The presence of CD103^−^ and CD103^+^ donor-derived CD8^+^ T cells in the transplanted SI mucosa was confirmed using chip cytometry (Figures 5A and S5C). Intestinal mucosa CD8^+^ T cells, both IELs and lamina propria lymphocytes (LPLs), had a predominantly effector memory (T_EM_) phenotype (Figures S5D and S5E), with CD8^+^ IELs dominated by CD103^+^ populations, consistent with prior work (Figures S5C and S5F) (Bartolomé-Casado et al., 2019). β2-integrin expression was constitutively high on all circulating memory T cells with greatest expression on T_EM_ CD8^+^ T cells (Figure S5F), as expected, because of the involvement of LFA-1 in tissue entry via ICAM-1 (Fagerholm et al., 2019).

The single-cell transcriptional data indicated that CD8^+^ CD69^+^CD103^+^ T cells from the intestinal mucosa formed a single transcriptional cluster; however, previous work has divided CD8^+^ CD103^+^ T cells into two populations based on their location in the epithelium or LPL (Bartolomé-Casado et al., 2019). We examined the phenotype of CD8^+^ CD103^+^ T cells in the IEL and LPL compartments in the healthy SI. Phenotypic differences between CD8^+^ CD103^−^ and CD103^+^ T cells were again observed in the LPL T cells, with greater expression of β2-integrin and KLRG1 on CD103^−^ cells and greater expression of CD161, CD7, and CD127 on CD103^+^ cells (Figure 5B). Chip cytometry of CD18, KLRG1, and granzyme K expression by CD103^−^ and CD103^+^ CD8^+^ T_RM_ cells *in situ* showed congruent expression to flow cytometry results (Figure 5C). The CD8^+^ CD103^+^ T cell populations in the IEL and LPL compartments showed comparable expression of β2-integrin, KLRG1, CD161, CD7, and CD127 to each other, bolstering the evidence that these populations are both transcriptionally and phenotypically similar, rather than representing distinct subsets (Figure 5B).

### CD103^+^ CD8^+^ intestinal T cells demonstrate greater capacity for cytokine production

**Figure 6:**
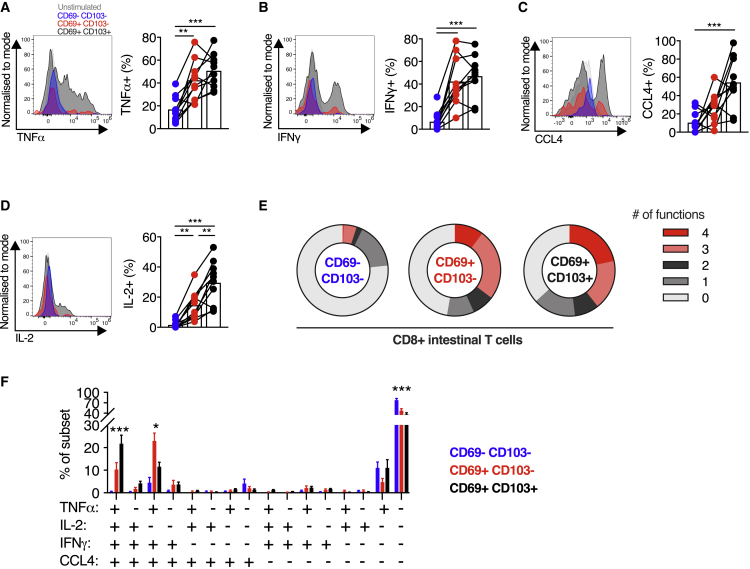
CD103^+^ CD8^+^ intestinal T cells demonstrate greater capacity for cytokine production (A–D) Cytokine production by small intestinal CD8^+^ T cells. Representative histograms of expression, and group summaries of proportion of CD8^+^ T cells expressing TNF-α (A), IFN-γ (B), CCL4 (C), and IL-2 (D) after 4 h stimulation with PMA and ionomycin in the presence of brefeldin A and monensin, categorized by CD69 and CD103 expression, in small intestinal biopsies from healthy control subjects (n = 10). (E) Mean proportion of CD8^+^ T cells expressing 0, 1, 2, 3, or 4 of the cytokines/chemokines TNF-α, IFN-γ, CCL4, and IL-2, categorized by CD69 and CD103 co-expression, from small intestinal biopsies from healthy control subjects (n = 10). (F) Mean percentage (± SEM) of CD8^+^ T cells co-expressing TNF-α, IFN-γ, CCL4, and/or IL-2 after PMA and ionomycin stimulation as described, categorized by CD69 and CD103 expression. Statistical analysis performed with one-way ANOVA with Tukey’s multiple-comparison test. ^∗^p ≤ 0.05, ^∗∗^p ≤ 0.01, ^∗∗∗^p ≤ 0.001.

To assess the cytokine production capacity of these subsets, SI T cells from healthy controls were stimulated for 4 h with phorbol 12-myristate 13-acetate (PMA) and ionomycin, before intracellular flow cytometry for tumor necrosis factor alpha (TNF-α), interferon gamma (IFN-γ), IL-2, CCL4, IL-17A, and IL-10; 77% of CD69^+^CD103^+^ CD8^+^ T cells produced at least one cytokine, a greater proportion than CD69^+^CD103^−^ CD8^+^ T cells (47.7% cytokine positive, p < 0.001) and CD69^−^ CD8^+^ T cells (37% cytokine positive, p < 0.001) produced (Figures 6A–6F). CD69^+^ populations produced more TNF-α and IFN-γ than CD69^−^ cells, irrespective of CD103 expression (Figures 6A and 6B). Although scRNA-seq data indicated increased *CCL4* transcripts in CD69^+^CD103^−^ CD8^+^ T cells, CCL4 production after stimulation was not different between the two CD69^+^ populations (Figure 6C). CD69^+^CD103^+^ CD8^+^ T cells expressed more IL-2 than either CD69^+^CD103^−^ or CD69^−^ populations (Figure 6D), consistent with a prior study of hepatic CD103^+^ CD8^+^ T_RM_ cells (Pallett et al., 2017).

There was a spectrum of functionality between the three populations, with CD69^−^ CD8^+^ T cells predominantly non- or mono-functional, CD69^+^CD103^+^ CD8^+^ T cells predominantly polyfunctional, and CD69^+^CD103^−^ CD8^+^ T cells showing intermediate functionality (Figures 6E and 6F), consistent with a prior report (Bartolomé-Casado et al., 2019). Quadruple functional cells were more common in the CD69^+^CD103^+^ population than in the CD69^+^CD103^−^ population (21.76% versus 10.32%, p < 0.0001) and were near absent in the CD69^−^ population (0.38%). These results demonstrate that the transcriptionally distinct CD103^−^ and CD103^+^ CD8^+^ T_RM_ cell populations differ functionally and phenotypically, with CD103^+^ CD8^+^ populations more polyfunctional and producing IL-2.

### CD103^−^ and CD103^+^ CD4^+^ T cells display analogous phenotypic and functional differences to their CD8^+^ counterparts

**Figure 7:**
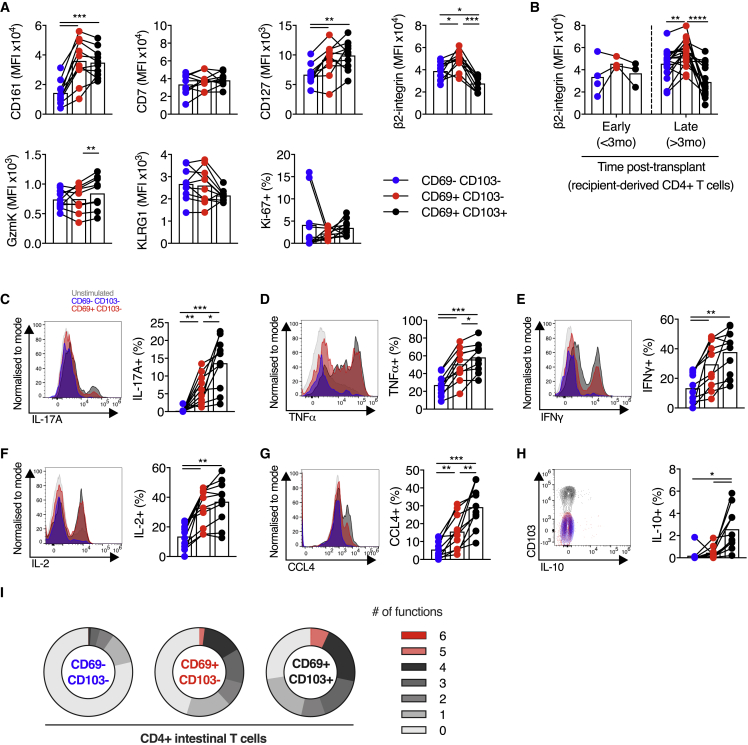
CD103^+^ and CD103^−^ CD4^+^ T cells display analogous phenotypic and functional differences to their CD8^+^ counterparts (A) MFI (CD161, CD7, CD127, β2-integrin, granzyme K, and KLRG1) or percentage positive (Ki-67) of CD4^+^ T cells, categorized by CD69 and CD103 expression, in small intestinal biopsies from healthy control subjects (n = 10). MFI represented by bars. Connecting lines represent populations from the same subject. Blue, CD69^−^CD103^−^ cells; red, CD69^+^CD103^−^ cells; black, CD69^+^CD103^+^ cells. (B) MFI of β2-integrin on recipient-derived CD4^+^ T cells, categorized by CD69 and CD103 expression and time after transplant, in intestinal transplant grafts (n = 21; 12 subjects). MFI represented by bars. (C–H) Cytokine production by small intestinal CD4^+^ T cells. Representative histograms of expression, and group summaries of proportion of CD4^+^ T cells expressing IL-17A (C), TNF-α (D), IFN-γ (E), IL-2 (F), CCL4 (G), and IL-10 (H) after 4 h stimulation with PMA and ionomycin in the presence of brefeldin A and monensin, categorized by CD69 and CD103 expression, in small intestinal biopsies from healthy control subjects (n = 10). (I) Mean proportion of CD4^+^ T cells expressing 0, 1, 2, 3, 4, 5, or 6 of the cytokines or chemokines IL-17A, TNF-α, IFN-γ, CCL4, IL-2, and IL-10, categorized by CD69 and CD103 co-expression, from small intestinal biopsies from healthy control subjects (n = 10). Statistical analysis performed with one-way ANOVA with Tukey’s multiple-comparison test. ^∗^p ≤ 0.05, ^∗∗^p ≤ 0.01, ^∗∗∗^p ≤ 0.001.

Despite not forming transcriptionally distinct clusters by scRNA-seq, CD4^+^ SI T cells differed in their phenotype, dependent on CD69 and CD103 expression. CD69^−^ populations expressed lower levels of CD161 and CD127, whereas CD69^+^CD103^−^ CD4^+^ T cells had more β2-integrin than either CD69^−^ or CD69^+^CD103^+^ populations had, analogous to their CD8^+^ counterparts (Figure 7A). Differences in the expression of CD7, KLRG1, and granzyme K between CD69^+^CD103^−^ and CD69^+^CD103^+^ CD8^+^ populations were not seen within the CD4^+^ populations. Recipient-derived, graft-infiltrating CD69^+^CD103^−^ CD4^+^ T cells also displayed more β2-integrin than their CD103^+^ counterparts did (mean fluorescence intensity [MFI], 51,753 versus 30,959, p < 0.0001; Figure 7B).

A donor-derived regulatory CD4^+^ FOXP3^+^ T cell population was detected in the scRNA-seq data (Figure 2C), and donor-derived CD25^+^CD127- CD4^+^ T cells were detected by flow cytometry in some subjects, consistent with potential long-term residency of SI CD4^+^ regulatory T cells (Figure S5H). Low cell numbers of this population precluded further analysis.

CD69^+^ CD4^+^ SI T cells were potent cytokine producers, as seen previously (Bartolomé-Casado et al., 2020) and demonstrated greater production of multiple cytokines upon short-term stimulation compared with CD69^−^ CD4^+^ T cells (Figures 7C–7I). In particular, IL-17A production was almost exclusively restricted to CD69^+^ T cells (Figure 7C). CD69^+^CD103^+^ CD4^+^ T cells demonstrated greater production of TNF-α, CCL4, IL-17A, and IL-10 than their CD69^+^CD103^−^ counterparts. There was a gradient of functionality between the three CD4^+^ populations, with CD103^+^ cells showing the greatest polyfunctional cytokine production (Figure 7I), as seen in CD8^+^ T cells. In summary, CD4^+^ SI T cell populations demonstrated analogous differences in phenotype and functionality to their CD8^+^ counterparts.

## Discussion

This study has identified two transcriptionally distinct states of functionally resident bona fide human intestinal CD8^+^ T_RM_ cells, which differ in phenotype and cytokine production. This was demonstrated using the rigorous approach of identifying donor-derived populations in the intestinal graft after transplant, which confirms functional tissue residency, and replicated in the healthy gut. The CD8^+^ T_RM_ cell subsets differ in the expression of several genes previously associated with human T_RM_ cells, which suggests that these signatures, derived from bulk RNA-seq data, may represent an amalgamation of transcriptionally distinct T_RM_ cell subsets (Kumar et al., 2017). Moreover, several TFs previously associated with residency programs were differentially expressed in intestinal T_RM_ cell clusters, suggesting potential mechanisms for the regulation of transcriptional heterogeneity.

The phenotypic differences between CD8^+^ T cell subsets were replicated within graft-infiltrating T cell populations, which are assumed to be establishing residency *de novo*. Although lateral migration of T_RM_ cells from recipient-derived intestinal tissue is a potential confounding mechanism, most intestinal transplant recipients have little or no remaining intestinal tissue after transplant, whereas murine work has demonstrated the limited motility of CD8^+^ T_RM_ cells in tissue (Thompson et al., 2019). Human studies of the development of residency over time and the clonal relatedness of T_RM_ cell subsets are a priority for future work.

αE-integrin is expressed on CD8^+^ T cells within both the IELs and a subset of LPLs, with previous work considering these to be distinct subsets (Bartolomé-Casado et al., 2019). However, the presence of only a single transcriptionally distinct CD69^+^CD103^+^ cluster, along with the phenotypic similarity between CD103^+^ IELs and LPLs, suggests that these represent a single subset within two spatially distinct compartments of the mucosa. Similarly, KLRG1 has been proposed as a marker for a distinct subset of CD103^−^ CD8^+^ T cells in the LPL (Bartolomé-Casado et al., 2019). However, the lack of sub-clusters within the CD69^+^CD103^−^ CD8^+^ T cell population in the single-cell data fails to support the conclusion that this is a transcriptionally distinct population.

The CD69^+^CD103^−^ CD8^+^ T_RM_ population, although definitively resident, as demonstrated by persistence after transplantation, is found at lower frequencies with time after transplant, making up a small proportion of T cells in the mucosa after 1 year post-transplant. This may be because the CD103^−^ population represents an intermediate state on the path to the CD103^+^ population or may be due to increased capacity for longevity and persistence in the CD103^+^ T_RM_ cell population. The two CD8^+^ T_RM_ cell populations do differ in IL-7R (CD127) expression, and in IL-2 production, which may indicate differing persistence and proliferative properties. Autocrine IL-2 production is critical to secondary proliferative responses and IFN-γ production in CD8^+^ T cells (Feau et al., 2011) and may be of particular relevance to IEL populations in which the infrequent CD4^+^ T cells may provide suboptimal help (Zimmerli et al., 2005).

In addition, the two CD8^+^ T_RM_ cell populations differ in the expression of chemokine receptors and integrins, indicating potential differences in tissue homing and retention. Of particular interest, β2-integrin is highly expressed on CD69^+^CD103^−^ T_RM_ cells, a population that remains difficult to positively distinguish from recent immigrants from the circulation, particularly in the context of inflammation, in which CD69 expression may represent cellular activation rather than residency. Despite constitutively high expression on peripheral CD8^+^ T_EM_ cells, β2-integrin expression is low on CD69^−^ T cells in intestinal tissue, suggesting that β2-integrin surface expression is reduced on recent tissue immigrants, before subsequent upregulation on CD69^+^CD103^−^ T_RM_ cells. β2-integrin, in combination with CD69 expression, could therefore represent an additional positive marker to identify the CD103^−^ T_RM_ cell population.

β2-integrin forms part of the heterodimer LFA-1, which facilitates firm adhesion on blood vessel endothelium via ICAM-1, a critical stage in lymphocyte trafficking (Fagerholm et al., 2019). β2-integrin also regulates the immunological synapse providing a co-stimulatory signal, both in the interaction with antigen-presenting cells and with infected target cells (Liu et al., 2009). LFA-1 is upregulated on liver-resident T cells and facilitates their patrolling of hepatic sinusoids, indicating a role for LFA-1 in T_RM_ cell motility and tissue surveillance (McNamara et al., 2017). β2-integrin and its interactions with ICAM-1 have been implicated in murine IEL development and function (Huleatt and Lefrançois, 1996). Intriguingly, the interaction of LFA-1 with ICAM-1 reduces cellular responses to tumor growth factor beta (TGF-β), a key cytokine in T_RM_ cell development (Verma et al., 2012), whereas TGF-β can inhibit LFA-1 expression and function (Boutet et al., 2016). This suggests a possible role for β2-integrin in the development of CD69^+^CD103^−^ CD8^+^ T_RM_ cells, which may be TGF-β-independent, as it is in mice (Bergsbaken and Bevan, 2015).

The *ITGB2* locus is a differentially methylated region in the blood and mucosa in IBD, and mucosal gene expression is associated with disease activity (Harris et al., 2014; Ventham et al., 2016; Román et al., 2013). We hypothesize that this association with IBD, in which T_RM_ cells have a key role (Zundler et al., 2019), could be mediated via altered β2-integrin expression and its effects on T_RM_ cells, or suggest a pathogenic role for one of the two T_RM_ cell subsets.

Single-cell transcriptional heterogeneity has previously been examined in lung T_RM_ cells, with two potential subsets identified, characterized by predominantly different gene signatures to those in our study (Snyder et al., 2019). However, the subsets contained both CD4^+^ and CD8^+^ cells co-clustering, which separated clearly in our study. Moreover, the lung T_RM_ cell subsets did not align with CD103 expression, in contrast to our work, and the transcriptional signatures of the clusters were also different. T_RM_ cell phenotype and behavior in the lung and in the intestine differ substantially (Thome et al., 2014), and this finding may be driven by such tissue-specific differences or may be an effect of the higher cell number in our study, allowing greater power to detect transcriptionally distinct sub-clusters. It remains unclear whether analogous subsets to the intestinal populations described in this study exist in other tissues, whether such subsets differ in residency characteristics and functional capacity, and whether they have distinct roles in human health and disease.

In conclusion, we have used the human model of intestinal transplantation to study single-cell heterogeneity in the donor-derived, bona fide intestinal T_RM_ cells. We found that CD8^+^ T_RM_ cells comprise two transcriptionally, phenotypically, and functionally distinct, subsets with parallel findings in CD4^+^ T cells. In particular, we report the association of β2-integrin expression with CD69^+^CD103^−^ intestinal T_RM_ cells. In addition to providing a useful marker for this population, β2-integrin could have a role in T_RM_ cell development and function.

## STAR★Methods

### Key Resources Table

**Table:** 

REAGENT or RESOURCE	SOURCE	IDENTIFIER
**Antibodies**		

Anti-human CD3 (clone OKT3) BV785	BioLegend	Cat# 317329
Anti-human CD3 (clone UCHT1) AF700	BioLegend	Cat# 300423
Anti-human CD3 (clone UCHT1) PerCP	BioLegend	Cat# 300427
Anti-human Vδ2 (clone B6) BV711	BioLegend	Cat# 331411
Anti-human CD4 (clone OKT4) BV650	BioLegend	Cat# 317435
Anti-human CD4 (clone SK3) BUV805	BD Biosciences	Cat# 612888
Anti-human CD4 (clone RPA-T4) PerCP	BioLegend	Cat# 300527
Anti-human Vα7.2 (clone 3C10) BV605	BioLegend	Cat# 351719
Anti-human CD45RA (clone HI100) BV785	BioLegend	Cat# 304139
Anti-human CD103 (clone Ber-ACT8) BV421	BioLegend	Cat# 350213
Anti-human CD103 (clone Ber-ACT8) APC-Cy7	BioLegend	Cat# 350227
Anti-human CD103 (clone Ber-ACT8) PE	BioLegend	Cat# 350205
Anti-human γδTCR (clone 11F2) FITC	Miltenyi Biotec	Cat# 130-114-029
Anti-human γδTCR (clone 11F2) VioBlue	Miltenyi Biotec	Cat# 130-113-507
Anti-human γδTCR (clone 5A6.E9) PE	Thermo Fisher Scientific	Cat# MHGD04
Anti-human β7-integrin (clone FIB504) PerCP-Cy5.5	BioLegend	Cat# 321219
Anti-human CD18 (clone L130) BUV563	BD Biosciences	Cat# 749441
Anti-human CD18 (clone 7E4) PE	Beckman Coulter	Cat# IM157OU
Anti-human CD45 (clone HI30) FITC	BioLegend	Cat# 304038
Anti-human CD69 (clone FN50) PE-Dazzle	BioLegend	Cat# 310941
Anti-human CD69 (clone FN50) BUV395	BD Biosciences	Cat# 564364
Anti-human CCR7 (clone G043H7) PE Dazzle	BioLegend	Cat# 353235
Anti-human CCR9 (clone L053E8) PE-Cy7	BioLegend	Cat# 358909
Anti-human CD8 (clone SK1) AF700	BioLegend	Cat# 344723
Anti-human CD8 (clone SK1) FITC	BioLegend	Cat# 344704
Anti-human CD8 (clone RPA-T8) AF532	Thermo Fisher Scientific	Cat# 58-0088-42
Anti-human CD161 (clone 191B8) APC	Miltenyi Biotec	Cat# 130-113-591
Anti-human CD161 (clone 191B8) FITC	Miltenyi Biotec	Cat# 130-113-592
Anti-human CD161 (clone 191B8) PE	Miltenyi Biotec	Cat# 130-092-677
Anti-human HLA-A3 (clone GAP.A3) PE	Thermo Fisher Scientific	Cat# 12-5754-41
Anti-human HLA-A2 (clone BB7.2) PE	BioLegend	Cat# 343306
Anti-human CD127 (clone A019D5) BV650	BioLegend	Cat# 351325
Anti-human CD127 (clone REA614) PE	Miltenyi Biotec	Cat# 130-113-976
Anti-human CD7 (clone M-T701) BB700	BD Biosciences	Cat# 566489
Anti-human KLRG1 (clone REA261) VioBlue	Miltenyi Biotec	Cat# 130-123-526
Anti-human KLRG1 (clone SA231A2) PE	BioLegend	Cat# 367711
Anti-human CCL4 (clone D21-1351) BV421	BD Biosciences	Cat# 562900
Anti-human Ki-67 (clone B56) BV480	BD Biosciences	Cat# 566172
Anti-human Granzyme B (clone GB11) BV510	BD Biosciences	Cat# 563388
Anti-human TNF-α (clone OKT3) BV650	BioLegend	Cat# 502935
Anti-human IL-17A (clone BL168) BV711	BioLegend	Cat# 512327
Anti-human IFN-γ (clone B27) BV750	BD Biosciences	Cat# 566357
Anti-human IL-2 (clone MQ1-17H12) PE-Dazzle	BioLegend	Cat# 500343
Anti-human Granzyme K (clone GM26E7) PE-Cy7	BioLegend	Cat# 370515
Anti-human Granzyme K (clone GM26E7) PE	BioLegend	Cat# 370511
Anti-human IL-10 (clone JES3-9D7) APC	BioLegend	Cat# 501409
Anti-human pan Cytokeratin (clone C-11) FITC	GeneTex	Cat# GTX11212
Anti-human Histone H3 (clone 17H2L9) AF488	Invitogen	Cat# MA702023
Near-IR Live-Dead amine-reactive dye	Thermo Fisher Scientific	Cat# L10119
Zombie Yellow Fixable Viability dye	BioLegend	Cat# 423103

**Biological samples**		

Leukocyte cones	NHS Blood and Transplant	https://www.nhsbt.nhs.uk
Intestinal biopsies (intestinal transplant recipients)	TGU Biobank	http://www.expmedndm.ox.ac.uk/tgu/tgu/home
Peripheral blood (intestinal transplant recipients)	TGU Biobank	As above
Intestinal biopsies (unaffected control subjects)	TGU Biobank	As above
Peripheral blood (unaffected control subjects)	TGU Biobank	As above

**Chemicals, peptides, and recombinant proteins**		

DNase I	Roche/ Merck	Cat# 11284932001
Percoll	GE Healthcare/ Merck	Cat# GE17-0891-01
LymphoPrep	Axis Shield	Cat# 07851
Cytofix/Cytoperm kit	BD Biosciences	Cat# 554714
Cytofix	BD Biosciences	Cat# 554655
DAPI (4’,6-Diamidino-2-Phenylindole, Dihydrochloride)	Thermo Fisher Scientific	Cat# D1306
Brillian Stain Buffer Plus	BD Biosciences	Cat# 566385

**Critical commercial assays**		

Cell Activation cocktail (without Brefeldin A)	Biolegend	Cat# 423301
Brefeldin A	Biolegend	Cat# 420601
Monensin	Biolegend	Cat# 420701
Chromium Single Cell 5′ Library & Gel Bead Kit	10x Genomics	Cat# PN-1000014
Chromium Single Cell 5′ Library Construction Kit	10x Genomics	Cat# PN-1000020
Chromium Single Cell V(D)J Enrichment Kit, Human T Cell	10x Genomics	Cat# PN-1000005
Chromium Single Cell A Chip Kit	10x Genomics	Cat# PN-1000009

**Deposited data**		

scRNA-seq (10x genomics) – 1 transplant donor - intestinal tissue resident memory T cells	This paper	GEO: GSE162687
scRNA-seq (Smartseq2) – 2 transplant donors – intestinal tissue resident memory T cells (CD103-/+)	This paper	GEO: GSE162687
scRNA-seq of ileal immune cells	Martin et al., 2019	https://scdissector.org/martin/
scRNA-seq of murine intestinal tissue resident memory T cells post-LCMV infection	Kurd et al., 2020	https://immunology.sciencemag.org/content/5/47/eaaz6894
scRNA-seq of colonic CD8+ T cells	Corridoni et al., 2020	GEO: GSE148837 / GSE148505

**Software and algorithms**		

Prism Version 8	GraphPad software	https://www.graphpad.com
ZellExplorer	Zellkraftwerk GmbH	http://www.zellkraftwerk.com/products/
FlowJo v9.9.5 & v10.6.1	FlowJo LLC	https://www.flowjo.com/
Cell Ranger v2.2.0	10x Genomics	https://www.10xgenomics.com
Seurat v2.3.4	(Butler et al., 2018); Stuart et al., 2019	https://satijalab.org/seurat/
Trimmomatic	Bolger et al., 2014	http://www.usadellab.org/
STAR v2.5.3a	Dobin et al., 2013	https://github.com/alexdobin/STAR
Samtools v1.6	Li et al., 2009	https://github.com/samtools/samtools
featureCounts v1.6.0	Liao et al., 2014	http://subread.sourceforge.net
Scater v1.10.1	McCarthy et al., 2017	https://www.bioconductor.org/packages/release/bioc/html/scater.html
Scran v1.12.1	Lun et al., 2016	https://bioconductor.org/packages/release/bioc/html/scran.html
M3Drop v3.10.4	Andrews et al., 2019	https://www.bioconductor.org/packages/release/bioc/html/M3Drop.html
SC3 v1.12.0	Kiselev et al., 2017	https://bioconductor.org/packages/release/bioc/html/SC3.html
DEsingle v1.2.1	Miao et al., 2018	https://bioconductor.org/packages/release/bioc/html/DEsingle.html
DAVID web tools	Huang et al., 2009	https://david.ncifcrf.gov/
Gene Set Enrichment Analysis v4.0.3	Subramanian et al., 2005	https://www.gsea-msigdb.org/gsea/

**Other**		

Zellsafe Tissue chips	Zellkraftwerk GmbH	Cat# 28050606/02-010
gentleMACS C Tubes	Miltenyi	Cat# 130-093-237

### Resource availability

#### Lead contact

Further information and requests for resources and reagents should be directed to and will be fulfilled by the Lead Contact, Paul Klenerman (paul.klenerman@medawar.ox.ac.uk).

#### Materials availability

This study did not generate new unique reagents.

#### Data and code availability

The single-cell RNA sequencing datasets generated during this study are available on the Gene Expression Omnibus (GEO) database (https://www.ncbi.nlm.nih.gov/geo/), accession number GEO: GSE162687.

### Experimental model and subject details

#### Human samples

Intestinal transplant recipients were identified via the Oxford University Hospitals NHS Foundation Trust (OUHFT) transplantation service (Oxford, United Kingdom). Healthy control study subjects were identified via the OUHFT endoscopy service at the time of routine endoscopy. Peripheral blood and intestinal biopsies were taken at the time of endoscopy under the study framework and consent of the Oxford Gastrointestinal Illnesses Biobank (REC Ref: 16/YH/0247). Patient and sample characteristics, including available information on gender, are described in Table S1. The influence of gender was not specifically considered in the analysis of study data, due to limited sample size.

Small intestinal biopsies were collected at the time of endoscopy and transported in R10 (RPMI-1640 [Lonza] + 10% FCS [Sigma-Aldrich] + 1% penicillin/streptomycin [Sigma-Aldrich]), before cryopreservation in freezing medium (90% FCS [Sigma-Aldrich], 10% DMSO [Sigma-Aldrich]). This method preserves immune cell viability, surface marker expression, and function (Konnikova et al., 2018). When required, samples were rapidly thawed in a 37°C water bath and washed in 20 mL R10 before tissue dissociation.

Duodenal samples were incubated in R10 media with 1 mg/ml Collagenase D (Roche) and 100 μg/ml DNase (Thermo Fisher Scientific) for one hour in a shaking incubator at 37°C. Biopsies were then dissociated by vigorous agitation using a GentleMACS Dissociator (Miltenyi Biotec), then strained through a 70 μm filter. Cells were washed with R10 media. For samples undergoing *ex vivo* stimulation or cell sorting, the mononuclear cells were isolated on a discontinuous 70% and 35% Percoll gradient (GE Healthcare) by centrifugation at 700 *g* for 20 minutes without brake. Mononuclear cells were collected from the interface and washed in R10.

For multiplex fluorescence chip cytometry, single intestinal biopsies were embedded in OCT cryo-embedding matrix (Thermo Fisher Scientific) then frozen in isopentane (Sigma-Aldrich) suspended over liquid nitrogen, and stored at −80°C until use.

Peripheral blood mononuclear cells (PBMCs) were isolated from subject blood samples by density gradient centrifugation. In brief, blood was diluted 1:1 with PBS, then layered onto Lymphoprep (Axis-Shield), and centrifuged at 973 *g* for 30 minutes without brake. The mononuclear layer was collected and washed with R10. Any remaining red blood cells were lysed with ACK (Ammonium-Chloride-Potassium) solution for 2-3 minutes, and washed again in R10, before cryopreservation in freezing medium as above.

### Method details

#### *Ex vivo* stimulation

*Ex vivo* stimulation was performed as previously described (Provine et al., 2018). In brief, purified intestine-derived mononuclear cells were plated at approximately 10^6^/well in a 96-well U-bottom plate. Cell stimulation cocktail containing phorbol 12-myristate 13-acetate (PMA) and ionomycin (BioLegend) was added in accordance with manufacturer’s instructions in the presence of brefeldin A and monensin (both BioLegend). Cells were incubated at 37°C, 5% CO_2_, for 4 hours.

#### Flow cytometry and cell sorting

For surface marker staining, cells were stained in 50 μL of FACS buffer (PBS + 1mM EDTA + 0.05% BSA) for 30 minutes at 4°C. Surface antibodies and clones used are listed in the Key resources table.

For intracellular cytokine staining, after surface staining as above, cells were fixed and permeabilised in 100 μL Cytofix/Cytoperm solution for 20 minutes at 4°C (BD Biosciences). Cells were then washed twice in Perm/Wash buffer (BD Biosciences). Intracellular staining was performed in 50 μL of Perm/Wash buffer (BD Biosciences) for 30 minutes at 4°C using antibodies and clones listed in the Key resources table.

After staining, cells were stored at 4°C protected from light until data acquisition. Flow cytometry data were acquired on a BD LSRII flow cytometer (BD Biosciences) or Aurora spectral flow cytometer (Cytek). For fluorescence-activated cell sorting (FACS) samples were surface stained as above, with DAPI (Thermo Fisher Scientific) used as viability dye. FACS was performed on an AriaIII (BD Biosciences; 70 μm nozzle). Antibodies were purchased from BioLegend, BD Biosciences, Miltenyi Biotec, or Thermo Fisher Scientific.

#### Chip cytometry

Samples were frozen in OCT (as described above), cryosectioned onto coverslips and placed in cytometer chips (Zellsafe Tissue chips, Zellkraftwerk, GmbH). Sections were fixed *in situ* at room temperature for 10 minutes using 4% paraformaldehyde, then washed with 10 mL PBS. Non-specific binding was blocked by incubating in 5% goat serum (Thermo Fisher Scientific) in PBS for one hour at room temperature. Fluorophore-conjugated antibodies (see Key resources table) were diluted for staining in PBS. Immunostaining was performed iteratively, with up to three colors applied simultaneously. Fluorophores were then photobleached and additional antibodies applied to build up the panel (Hennig et al., 2009). Images were acquired using a Zellscanner One Chip cytometer (Zellkraftwerk) and ZellExplorer software.

#### 10× Genomics library preparation and sequencing

scRNA-seq libraries were generated using 10x Genomics Chromium Single Cell V(D)J Reagents Kits (v1 Chemistry) following manufacturer’s instructions. Cells were resuspended in PBS with 0.04% BSA at ∼1000 cells/μL and loaded onto a single lane of the Chromium Controller. Captured cell number was 1,774. Library quality and concentration was determined using a TapeStation (Agilent) and Qubit 2.0 Fluorometer (Thermo Fisher Scientific). Libraries were sequenced on an Illumina HiSeq 4000 as per manufacturer’s instructions to a mean depth of 63,471 reads/cell. Library generation and sequencing were performed at the Oxford Genomics Centre (Wellcome Centre for Human Genetics, University of Oxford).

#### Smart-Seq2 library preparation and sequencing

Single cells were index-sorted into 96-well plates with one cell per well in 2.3 μL lysis buffer (0.8% (vol/vol) Triton X-100 and 2 U/μL RNase inhibitor). Smart-Seq2 libraries were generated following the published protocol with External RNA Controls Consortium (ERCC) RNA (1:100,000) added prior to sequencing (Picelli et al., 2014). cDNA was pre-amplified by PCR (21 cycles). Libraries were sequenced on an Illumina HiSeq 4000 with 75 base pair paired-end reads. Library generation and sequencing were performed at the Oxford Genomics Centre (Wellcome Centre for Human Genetics, University of Oxford).

### Quantification and statistical analysis

#### Graphs and statistical analyses

All statistical analyses and graphs, except transcriptional data, were performed using GraphPad Prism Software Version 8 (La Jolla, CA). Details of specific statistical tests used to determine statistical significance are found in the relevant figure legends. All data are presented as mean ± SEM unless stated otherwise.

#### Flow cytometry data analysis and statistics

Flow cytometry data were analyzed using FlowJo version 9.9.5 and version 10.6.1 (FlowJo, LLC). For phenotypic analysis of rare cell subsets, populations with fewer than 10 cells were excluded.

#### Droplet-based (10× Genomics) scRNA-seq data analysis

FastQ generation, read alignment, barcode counting, and unique molecular identifier (UMI) counting were performed using the Cell Ranger pipeline v2.2.0. Downstream processing steps were performed using Seurat v2.3.4 (Butler et al., 2018; Stuart et al., 2019). Briefly, TCR and BCR genes, and genes expressed in fewer than 10 cells, were removed. Cells with < 3,460 UMIs (local minimum of the UMI distribution to the left of the mode UMI count), < 500 genes, and > 10,000 UMIs, > 2,500 genes, and/or > 10% mitochondrial reads were removed (Figure S2A). Variable genes were identified using M3Drop (Andrews et al., 2019). Data were log normalized and scaled, with cell-cell variation due to UMI counts, percent mitochondrial reads, and S and G2M cell cycle scores regressed out. The top 10 principal components (PCs) were used as input for graph-based clustering (0.4 resolution), as determined by visual inspection of the scree plot. Clusters were visualized by tSNE. Differential gene expression analysis between clusters was performed using the Wilcoxon Rank Sum test (FindMarkers function with default parameters).

#### TCR clonotype analysis (10× Genomics)

Single-cell V(D)J sequences and annotations were generated using Cell Ranger v2.2.0. The filtered_contig_annotations.csv output file from cellranger vdj was filtered to retain only full-length, productive contigs associated with the TCR⍺ or TCRβ chain. Cells were filtered to retain only live cells (as determined by gene expression analysis), and to remove cells lacking a TCR⍺ or TCRβ chain, expressing two TCR⍺ and two TCRβ chains, or expressing more than two TCR⍺ or TCRβ chains. TCR clonotypes were defined as cells with identical TCR segment usage, and CDR3⍺ and CDR3β nucleotide sequences. Assuming some TCR chain dropout, clonotypes were allowed to contain a mixture of cells with a single or two TCR⍺ (or TCRβ) chains, so long as all detected chains exactly matched those within the clonotype.

#### Plate-based (Smart-Seq2) scRNA-seq data analysis

Reads were trimmed to remove contaminating adaptor and oligo-dT primer sequences using Trimmomatic v0.36 (Bolger et al., 2014). Trimmed reads were aligned to the human genome (hg38 assembly) plus added ERCC “spike-in” sequences using STAR v2.5.3a (--outFilterMismatchNoverLmax 0.04 --outFilterType BySJout --outMultimapperOrder Random) (Dobin et al., 2013). Alignments were filtered using Samtools v1.6 (Li et al., 2009) to retain only primary alignments and properly paired reads. Ensembl gene counts were generated using featureCounts v1.6.0 (-C -B -p) (Liao et al., 2014). Poor quality cells that fit one or more of the following criteria were removed from the analysis (Figures S2G–S2I): small log-library size (< 3 median absolute deviations [MADs] below the median), low percentage of uniquely mapped reads (< 55%), low gene count (< 3 MADs below the median), high percentage of ERCCs (> 37.5%), or high mitochondrial read fraction (> 6%). Outlier cells with a large library size or high gene count (potential doublets) were also removed. Genes with “undetectable” expression were removed (gene defined as “detectable” if at least five read counts in two cells), along with TCR (and BCR) genes. Log-normalized expression values were generated using the normalize function from scater v1.10.1 (McCarthy et al., 2017) with cell-specific size factors calculated using scran v1.12.1 (Lun et al., 2016). Feature selection was performed using M3Drop v3.10.4 (FDR < 0.01) (Andrews et al., 2019) and the expression matrix subset to retain only the selected genes. Clustering analysis was performed using SC3 v1.12.0 (Kiselev et al., 2017). Clusters were visualized by UMAP generated using the top 30 PCs (McInnes et al., 2018). Differential gene expression analysis was performed using DEsingle v1.2.1 (Miao et al., 2018) (FDR < 0.05).

#### Data integration with published datasets

For comparison of the T_RM_ gene sets with an external non-transplant human dataset (Martin et al., 2019), the web-based graphical interface for the data was used (https://scdissector.org/martin). Ileal T cell clusters with high expression of CD8A and CD8B, and low expression of CD4, were chosen for analysis. Consensus DEGs between populations 1 and 2 identified in both scRNA-seq datasets (Figure 3D) were examined, with hierarchical clustering of the expression of genes performed.

For analysis of the scRNA-seq data of colonic CD8+ T cells from healthy and ulcerative colitis patients, published in Corridoni et al. (2020), the authors supplied us with a Seurat object containing the data presented in Figure 4B of their study. The resting, conventional CD8+ T cell clusters were retained for analysis: FGFBP2+, GZMK+ (1), GZMK+ (2), TRM, Memory, IELs, IL26+. Hierarchical clustering was performed using the average expression of all genes to establish the relationship between these seven clusters. A heatmap was generated showing the average expression of the 30 DEGs between CD103+ and CD103- T_RM_ cells that coincided between experiment 1 and experiment 2.

For Gene Set Enrichment Analysis (GSEA) within DEGs between murine intestinal T_RM_ cell clusters post-LCMV infection (Kurd et al., 2020), a gene set associated with population 2/CD103- T_RM_ cells identified in this study was created. This included all genes differentially expressed in either experiment, and showing congruent direction of fold change in both experiments. These genes were converted to murine orthologs using DAVID Bioinformatics Resources 6.7 (https://david.ncifcrf.gov/) (Huang et al., 2009) and Mouse Genome Database (MGD) at the Mouse Genome Informatics website (http://www.informatics.jax.org). GSEA was performed using GSEA v4.0.3 (http://www.gsea-msigdb.org/gsea) (Subramanian et al., 2005).

#### Identification of TF regulons

TF regulons were identified using the SCENIC workflow (Aibar et al., 2017; Van de Sande et al., 2020). Briefly, the single-cell log-normalized gene expression matrix was further filtered to remove genes expressed in fewer than 1% of cells or with a total raw count of less than 30 UMIs. Potential target genes for a supplied list of human TFs (Lambert et al., 2018) were identified based on co-expression using the GRNBoost2 algorithm (Moerman et al., 2019). Co-expression modules were filtered using cis-regulatory motif analysis (RcisTarget) to retain only modules enriched for putative direct-binding targets of the corresponding TF, and remaining modules were pruned to remove targets lacking motif support. Where multiple modules were identified for a TF, these were combined to result in a single regulon per TF. Finally, cells were scored for the activity of each TF regulon using the AUCell algorithm. The Wilcoxon Rank Sum test (FindMarkers function, Seurat R package) was used to identify regulons with a statistically increased AUCell score in a given cluster relative to all other clusters.

## Supplemental information

Document S1. Figures S1–S5

Table S1. Subject and sample characteristics, related to STAR methods

Table S2. DEGs between CD8^+^ population 1 and CD8^+^ population 2 from experiment 1, related to Figure 2 • log2FC = log_2_ fold change of average expression between CD8+ population 1 and CD8+ population 2 • adj p-value = Bonferroni corrected p-value

Table S3. Differentially expressed genes between clusters 2 + 3 and cluster 1 from experiment 2, related to Figure 3 • log2FC = log_2_ fold change of average expression between clusters 2 + 3 and cluster 1 • adj p-value = Benjamini-Hochberg corrected p-value

Table S4. Cluster-specific transcription factor regulons from experiment 1, related to Figure 3 • log2FC = log2 fold change of average AUCell score between cluster and all other cells • adj p-value = Bonferroni corrected p-value

Document S2. Article plus supplemental information
