# Supplementary material for: Human intestinal tissue-resident memory T cells comprise transcriptionally and functionally distinct subsets
Source: Cell Rep. 2021 Jan 19;34(3):108661. doi: 10.1016/j.celrep.2020.108661 (PMC7816164; doi:10.1016/j.celrep.2020.108661)
Supplement: Document S1. Figures S1–S5 [file mmc1.pdf]

Cell Reports, Volume *34*

Supplemental Information

Human intestinal tissue-resident memory T cells comprise transcriptionally and functionally distinct subsets

Michael E.B. FitzPatrick, Nicholas M. Provine, Lucy C. Garner, Kate Powell, Ali Amini, Sophie L. Irwin, Helen Ferry, Tim Ambrose, Peter Friend, Georgios Vrakas, Srikanth Reddy, Elizabeth Soilleux, Paul Klenerman, and Philip J. Allan

Supplemental material

Figures (below):

- **Figure S1**
- **Figure S2**
- **Figure S3**
- **Figure S4**
- **Figure S5**

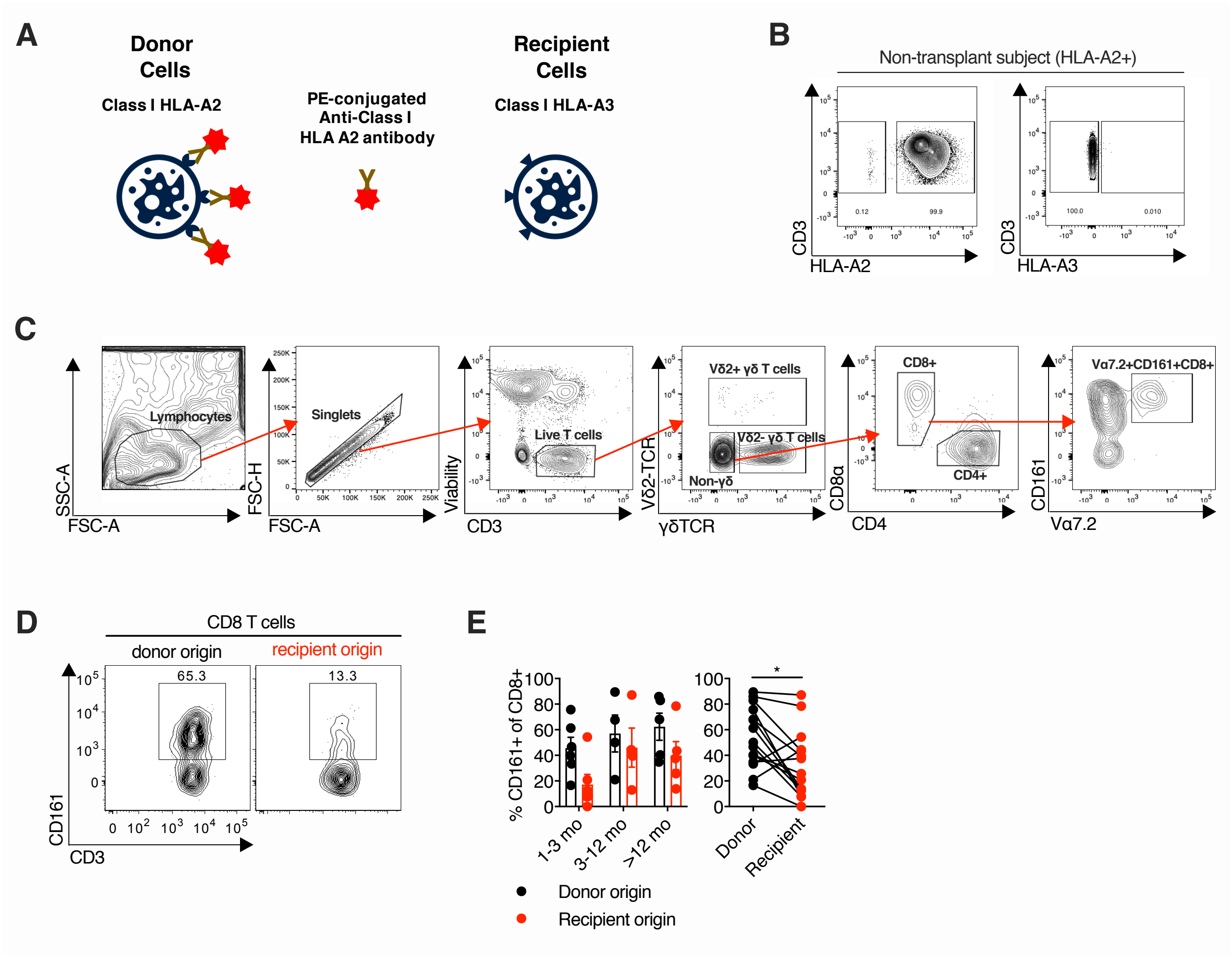

Figure S1 (related to Figure 1).

(A) Illustration demonstrating the principle of HLA allele congenic cell tracking to identify donor- and recipient-derived T cell populations in the transplanted small intestinal graft using fluorophore-conjugated antibodies to Class I HLA haplotypes discordant between donor and recipient.

(B) Representative flow cytometry plot of Class I HLA non-specific staining (0.01-0.12%) from a non-transplant PBMC sample. Gated on live CD3+ T cells.

(C) Representative flow cytometry gating scheme for identification of T cell subsets.

(D) Representative flow cytometry plot of CD161 expression on paired donor- and recipient-derived CD8+ intestinal T cells.

(E) Percentage of CD161+ donor- and recipient-derived CD8+ intestinal T cells categorised by time post-transplant (left), or grouped, with paired samples connected by black lines (right) (n=15 (8 subjects); mean +/- SEM).

Statistical analysis performed with Wilcoxon matched-pairs signed rank test. * $P \leq 0.05$.

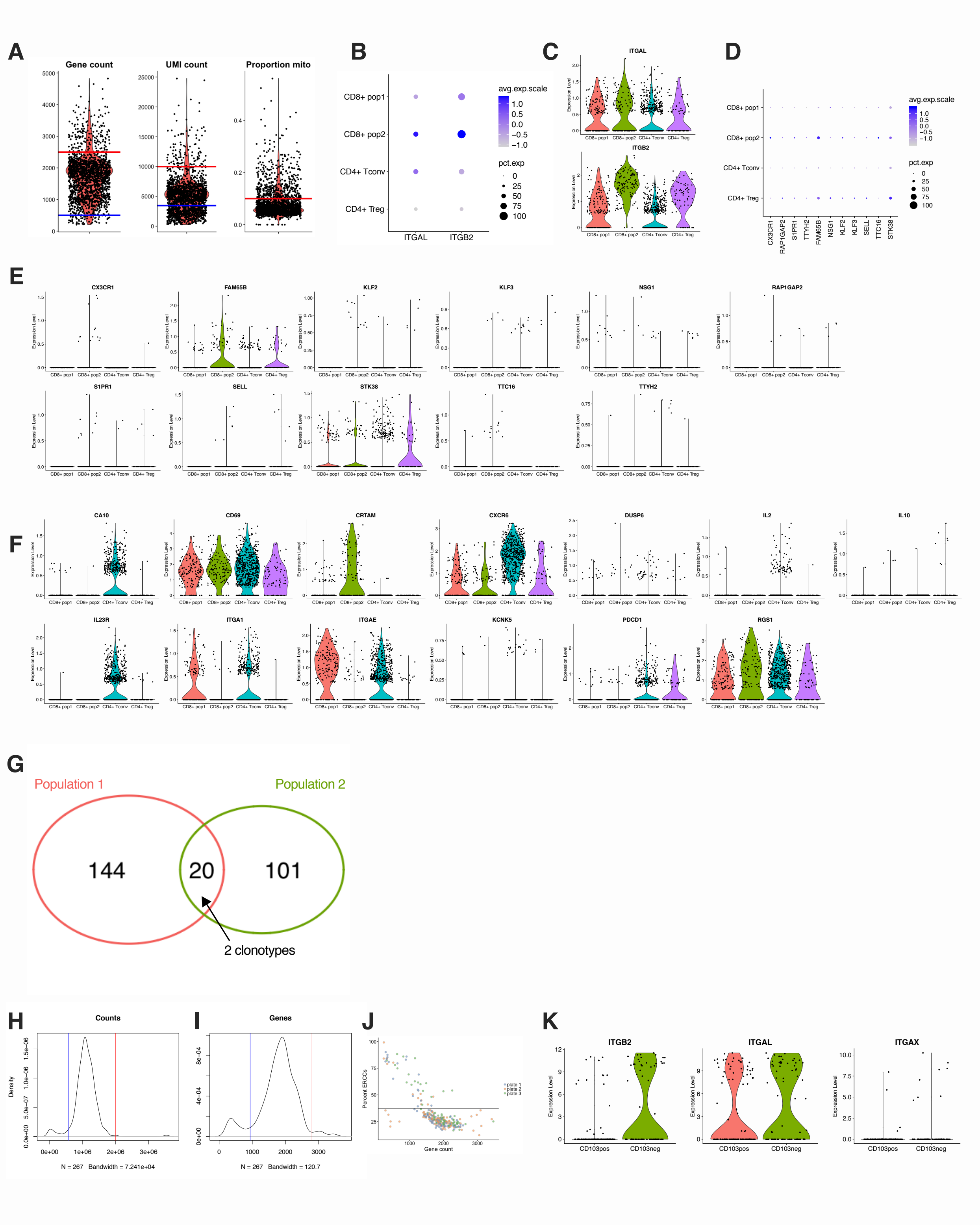
A
Gene count
UMI count
Proportion mito
B
CD8+ pop1
CD8+ pop2
CD4+ Tconv
CD4+ Treg
ITGAL
ITGB2
avg.exp.scale
pct.exp
C
ITGAL
ITGB2
Expression Level
D
CX3CR1
RAP1GAP2
S1PR1
TTYH2
FAM65B
NSG1
KLF2
KLF3
SELL
TTC16
STK38
E
F
CA10
CD69
CRTAM
CXCR6
DUSP6
IL2
IL10
IL23R
ITGA1
ITGAE
KCNK5
PDCD1
RGS1
G
Population 1
Population 2
144
20
101
2 clonotypes
H
Counts
Density
N = 267 Bandwidth = 7.241e+04
I
Genes
N = 267 Bandwidth = 120.7
J
Percent ERCCs
Gene count
plate 1
plate 2
plate 3
K
ITGB2
ITGAL
ITGAX
CD103pos
CD103neg

Figure S2 (related to Figure 2 and 3).
(A) Quality control parameters and thresholds for 10x Genomics scRNAseq Experiment 1. Number of genes per cell (left), number of unique molecular identifiers (UMIs) per cell (centre), and percentage of mitochondrial reads per cell (right) are displayed. Each dot represents a single cell, with red and blue lines indicating maximum and minimum thresholds, respectively.
(B) Dot plot showing expression of *ITGB2* and its potential heterodimeric partner *ITGAL* in Experiment 1. Other potential heterodimeric alpha integrin partners, *ITGAD*, *ITGAM*, and *ITGAX* were not detected. Dot size indicates the proportion of cells in which the gene is expressed. Colour intensity indicates the mean expression level of the gene.
(C) Violin plots showing expression of *ITGB2* and its potential heterodimeric partner *ITGAL* in Experiment 1. Other potential heterodimeric alpha integrin partners, *ITGAD*, *ITGAM*, and *ITGAX* were not detected.
(D) Dot plot showing expression in Experiment 1 of 11 genes previously negatively associated with tissue residency (downregulated in human CD69+ cells in comparison to CD69- T cells (Kumar et al., 2017)). Dot size indicates the proportion of cells in which the gene is expressed. Colour intensity indicates the mean expression level of the gene. Other genes in the gene set were not detected in the data (*SBK1*, *NPDC1*, *KRT72*, *SOX13*, *KRT73*, *TSPAN18*, *PTGDS*).
(E) Violin plots showing expression in Experiment 1 of 11 genes previously negatively associated with tissue residency (downregulated in human CD69+ cells in comparison to CD69- T cells (Kumar et al., 2017)).
(F) Violin plots showing expression in Experiment 1 of 13 genes previously associated with tissue residency in human CD69+ T cells (Kumar et al., 2017), demonstrating variable expression in conventional T cell clusters.
(G) Venn diagram showing the number of cells with TCR clonotypes unique to population 1 or 2, or shared between population 1 and 2 in Experiment 1.
(H-J) Quality control parameters and thresholds for Smart-Seq2 scRNAseq Experiment 2. (H) Histogram of number of reads per cell and (I) number of genes per cell. Red and blue lines indicate maximum and minimum thresholds, respectively. (J) Plot showing the percentage of External RNA Controls Consortium (ERCC) spike-ins per cell against the number of genes per cell. Black line indicates upper threshold.
(K) Violin plots showing expression of *ITGB2* and its potential heterodimeric partners *ITGAL* and *ITGAX* in Experiment 2. *ITGAD* and *ITGAM* were not detected in the data.

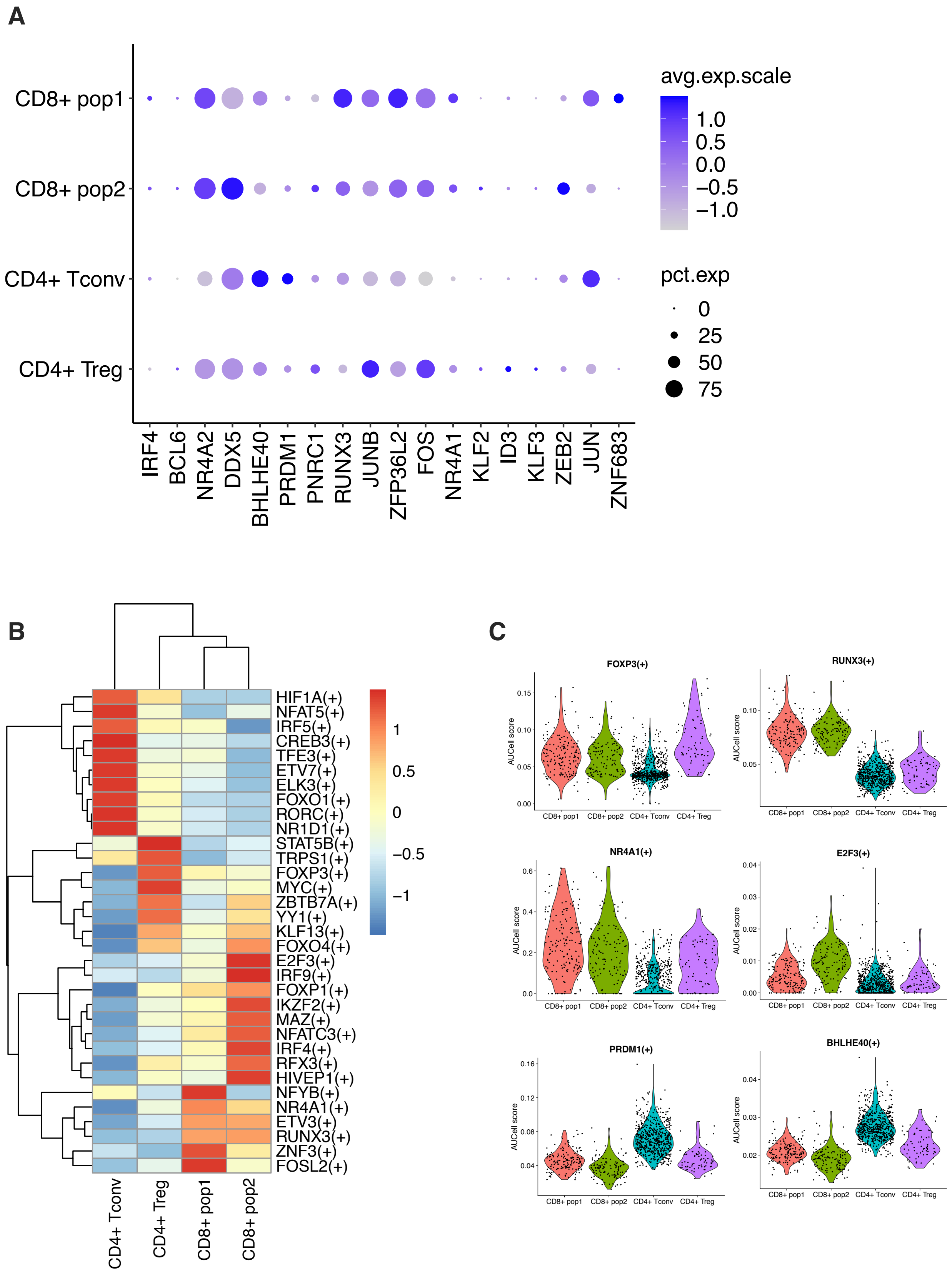
A
CD8+ pop1
CD8+ pop2
CD4+ Tconv
CD4+ Treg
avg.exp.scale
1.0
0.5
0.0
−0.5
−1.0
pct.exp
0
25
50
75
IRF4
BCL6
NR4A2
DDX5
BHLHE40
PRDM1
PNRC1
RUNX3
JUNB
ZFP36L2
FOS
NR4A1
KLF2
ID3
KLF3
ZEB2
JUN
ZNF683
B
HIF1A(+)
NFAT5(+)
IRF5(+)
CREB3(+)
TFE3(+)
ETV7(+)
ELK3(+)
FOXO1(+)
RORC(+)
NR1D1(+)
STAT5B(+)
TRPS1(+)
FOXP3(+)
MYC(+)
ZBTB7A(+)
YY1(+)
KLF13(+)
FOXO4(+)
E2F3(+)
IRF9(+)
FOXP1(+)
IKZF2(+)
MAZ(+)
NFATC3(+)
IRF4(+)
RFX3(+)
HIVEP1(+)
NFYB(+)
NR4A1(+)
ETV3(+)
RUNX3(+)
ZNF3(+)
FOSL2(+)
1
0.5
0
−0.5
−1
CD4+ Tconv
CD4+ Treg
CD8+ pop1
CD8+ pop2
C
FOXP3(+)
RUNX3(+)
NR4A1(+)
E2F3(+)
PRDM1(+)
BHLHE40(+)
AUCell score
CD8+ pop1
CD8+ pop2
CD4+ Tconv
CD4+ Treg

Figure S3 (related to Figures 2 and 3).
(A) Dot plot showing expression of a curated list of TFs linked to tissue residency in prior publications in T_{RM} cell clusters in Experiment 1. Dot size indicates the proportion of cells in which the gene is expressed. Colour intensity indicates the mean expression level of the gene.
(B) Heatmap showing the activity of TF regulons (gene sets predicted to be regulated by a given TF) in T_{RM} cell clusters in Experiment 1. The top 10 regulons with significantly increased activity (FDR<0.001) in each cluster are shown.
(C) Violin plots showing the activity of FOXP3, RUNX3, NR4A1, E2F3, PRDM1, and BHLHE40 regulons in T_{RM} cell clusters in Experiment 1.

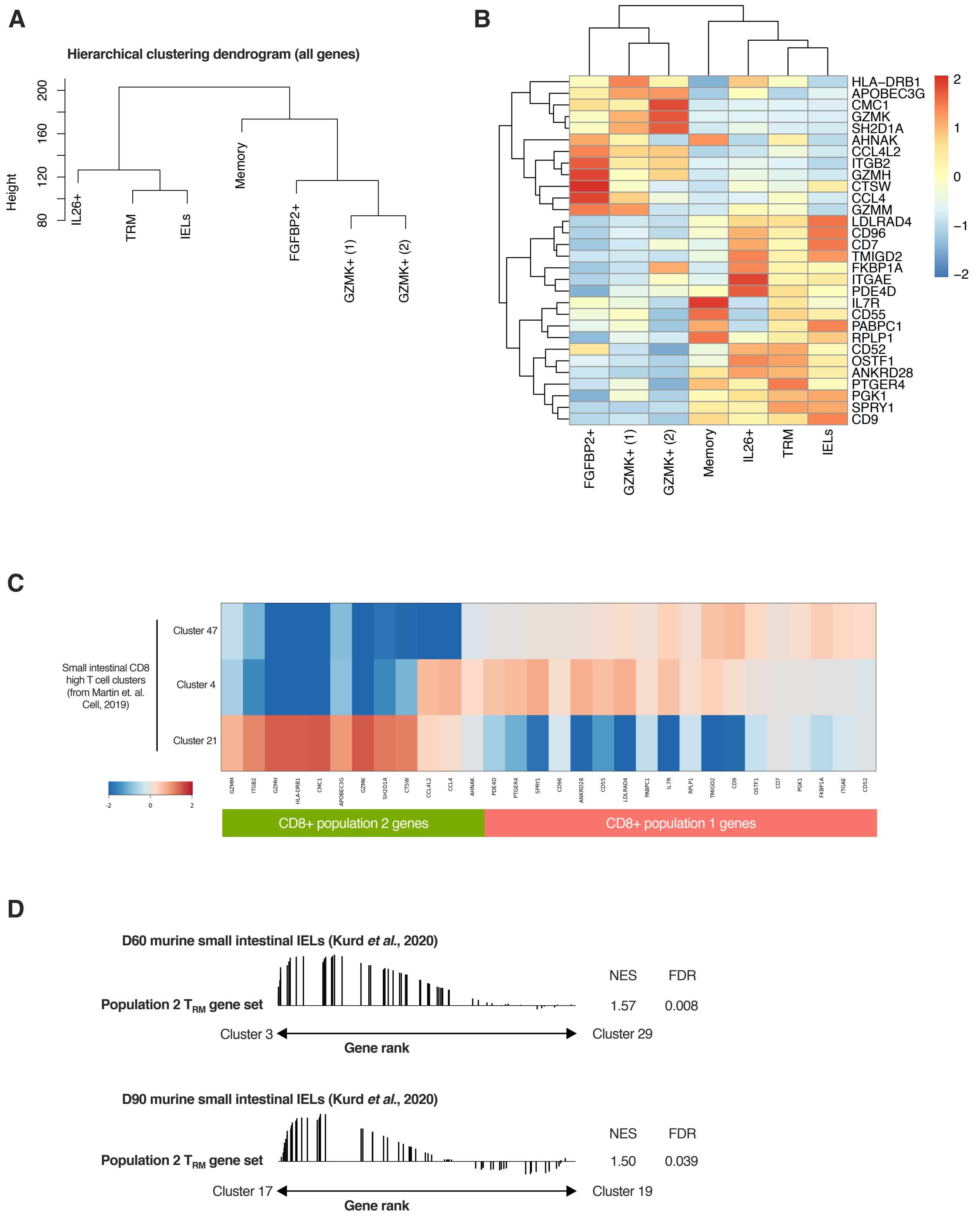
A
Hierarchical clustering dendrogram (all genes)
Height
80
120
160
200
IL26+
TRM
IELs
Memory
FGFBP2+
GZMK+ (1)
GZMK+ (2)
B
HLA−DRB1
APOBEC3G
CMC1
GZMK
SH2D1A
AHNAK
CCL4L2
ITGB2
GZMH
CTSW
CCL4
GZMM
LDLRAD4
CD96
CD7
TMIGD2
FKBP1A
ITGAE
PDE4D
IL7R
CD55
PABPC1
RPLP1
CD52
OSTF1
ANKRD28
PTGER4
PGK1
SPRY1
CD9
2
1
0
−1
−2
FGFBP2+
GZMK+ (1)
GZMK+ (2)
Memory
IL26+
TRM
IELs
C
Small intestinal CD8 high T cell clusters (from Martin et. al. Cell, 2019)
Cluster 47
Cluster 4
Cluster 21
-2
0
2
CD8+ population 2 genes
CD8+ population 1 genes
D
D60 murine small intestinal IELs (Kurd et al., 2020)
Population 2 TRM gene set
NES
FDR
1.57
0.008
Cluster 3
Cluster 29
Gene rank
D90 murine small intestinal IELs (Kurd et al., 2020)
Population 2 TRM gene set
NES
FDR
1.50
0.039
Cluster 17
Cluster 19
Gene rank

Figure S4 (related to Figure 3).
(A-B) Examination of transcriptional profiles of conventional CD8+ intestinal T cells from human colon samples (published in (Corridoni et al., 2020)). (A) Hierarchical clustering of colonic CD8+ T cell clusters based on total gene expression. (B) Heatmap of expression of the T_{RM} cell subset transcriptional signature within CD8+ colonic T cells.
(C) Normalized expression of the T_{RM} cell subset transcriptional signature within CD8A/CD8B^{hi} and CD4^{lo} intestinal T cell clusters from human ileum samples (published in (Martin et al., 2019)).
(D) Gene Set Enrichment Analysis showing the enrichment of a gene set associated with human CD103- T_{RM} cells from this study in clusters of murine intestinal T_{RM} cells at day 60 (D60) and day 90 (D90) post-LCMV infection. NES, normalized enrichment score. FDR, false discovery rate (<0.25 considered significant).

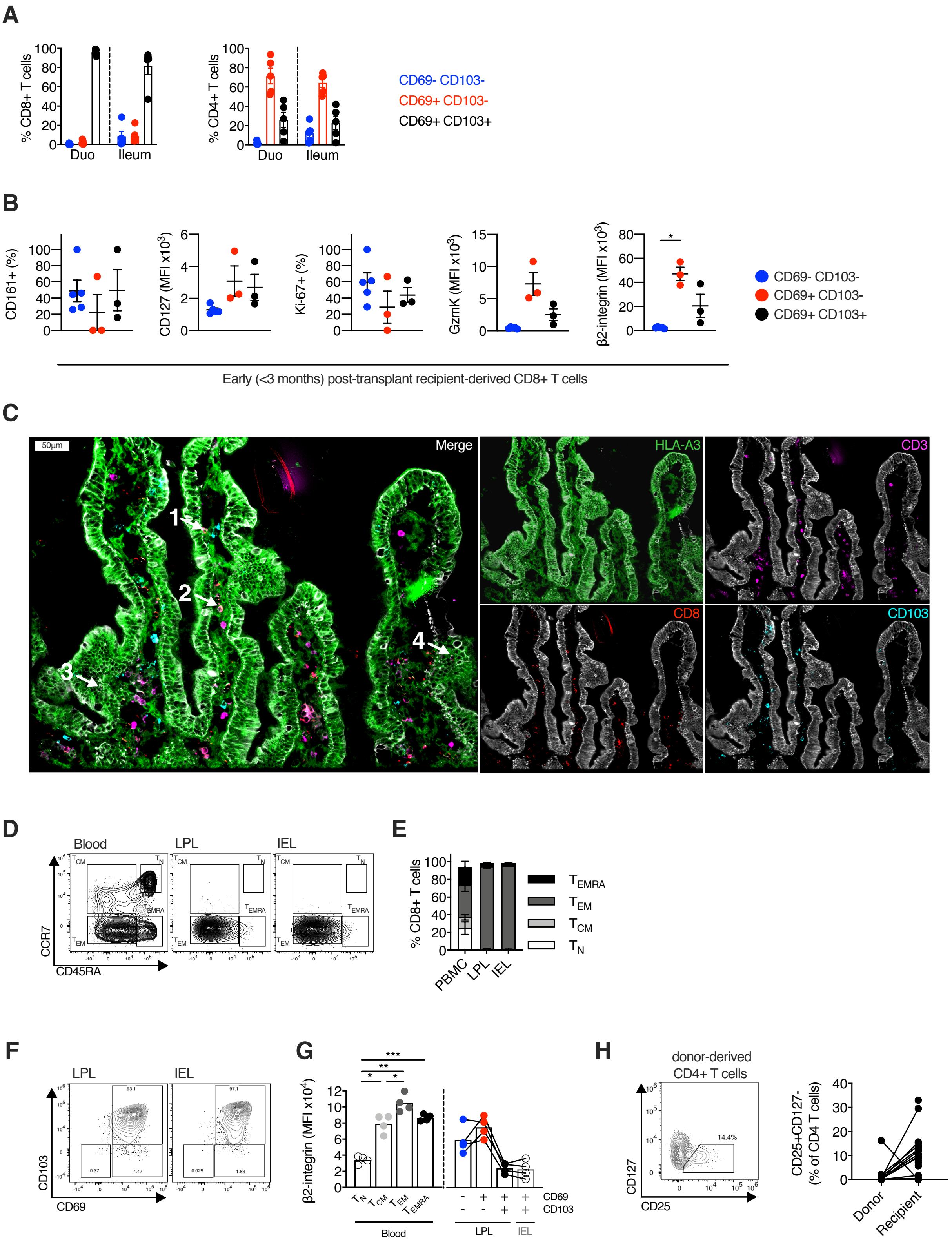
A
% CD8+ T cells
Duo
Ileum
% CD4+ T cells
CD69- CD103-
CD69+ CD103-
CD69+ CD103+
B
CD161+ (%)
CD127 (MFI x10^3)
Ki-67+ (%)
GzmK (MFI x10^3)
β2-integrin (MFI x10^3)
Early (<3 months) post-transplant recipient-derived CD8+ T cells
C
50µm
Merge
HLA-A3
CD3
CD8
CD103
D
Blood
LPL
IEL
CCR7
CD45RA
E
% CD8+ T cells
PBMC
LPL
IEL
TEMRA
TEM
TCM
TN
F
CD103
CD69
G
β2-integrin (MFI x10^4)
Blood
CD69
CD103
H
donor-derived
CD4+ T cells
14.4%
CD127
CD25
CD25+CD127-
(% of CD4 T cells)
Donor
Recipient

Figure S5 (related to Figure 4 and 5).
(A) The proportion of CD8+ T cells co-expressing CD69 and CD103 in small intestinal biopsies from the duodenum (Duo) or ileum from healthy control subjects (n=5 for both duodenum and ileum).
(B) Phenotypic analysis of recipient-derived CD8+ T cell populations infiltrating the intestinal graft in the early post-transplant period (<3 months). Proportion of CD161+ cells, Ki-67+ cells or MFI of CD127, granzyme K, and β2-integrin of recipient-derived CD8+ T cells, categorised by CD69 and CD103 expression, in intestinal transplant grafts at early (<3 months) timepoints post-transplantation (n=5; 2 subjects). Mean percentage or MFI represented by bars. Black lines connect populations from the same sample.
(C) Fluorescence microscopy chip cytometry image from a separate section from the same donor as Fig. 5A. False colour fluorescence imaging for cytokeratin (grey), CD3 (purple), CD8 (red), CD103 (blue), and HLA-A3 (green). Representative donor-derived CD8+ CD103+ and CD103- cells for further characterization (Fig. 5C) are numbered.
(D-G) CD8+ T cell memory status and phenotype in intestinal epithelium and peripheral blood. (D) Representative plot of CCR7 and CD45RA expression of circulating, lamina propria lymphocyte (LPL), and intra-epithelial lymphocyte (IEL) CD8+ T cells, identifying naïve (T_N), central memory (T_{CM}), effector memory (T_{EM}), and terminally differentiated effector memory (T_{EMRA}) populations. (E) Stacked plots showing mean percentage (+/- SEM) of CD8+ T cells comprising naïve (T_N; white), central memory (T_{CM}; light grey), effector memory (T_{EM}; dark grey), and terminally differentiated effector memory (T_{EMRA}; black) populations (n=4). (F) Representative flow cytometry plot of CD69 and CD103 expression on LPL and IEL T cells. (G) MFI of β2-integrin expression on circulating, LPL, and IEL CD8+ T cells (n=4). Mean percentage or MFI represented by bars. Connecting lines represent populations from the same subject.
(H) Representative flow cytometry gating of CD4+ CD25+CD127lo T cells. Percentage of donor- or recipient-derived intestinal CD4+ CD25+CD127lo T cells. Connecting lines represent populations from the same subject (n=23).
Statistical analysis performed with one-way ANOVA with Tukey's multiple comparison test. * $P \leq 0.05$; ** $P \leq 0.01$; *** $P \leq 0.001$; **** $P \leq 0.0001$.
